# Insights into the Physiology and Ecology of the Brackish-Water-Adapted Cyanobacterium *Nodularia spumigena* CCY9414 Based on a Genome-Transcriptome Analysis

**DOI:** 10.1371/journal.pone.0060224

**Published:** 2013-03-28

**Authors:** Björn Voß, Henk Bolhuis, David P. Fewer, Matthias Kopf, Fred Möke, Fabian Haas, Rehab El-Shehawy, Paul Hayes, Birgitta Bergman, Kaarina Sivonen, Elke Dittmann, Dave J. Scanlan, Martin Hagemann, Lucas J. Stal, Wolfgang R. Hess

**Affiliations:** 1 Genetics and Experimental Bioinformatics Group, Faculty of Biology, University of Freiburg, Freiburg, Germany; 2 Department of Marine Microbiology, Royal Netherlands Institute of Sea Research, Yerseke, The Netherlands; 3 Food and Environmental Sciences, Division of Microbiology, Viikki Biocenter, University of Helsinki, Helsinki, Finland; 4 Plant Physiology, Institute Biosciences, University of Rostock, Rostock, Germany; 5 IMDEA-Agua, Alcalá de Henares, Madrid, Spain; 6 Faculty of Science, University of Portsmouth, Portsmouth, United Kingdom; 7 Department of Botany, Stockholm University, Stockholm, Sweden; 8 Institute for Biochemistry and Biology, University of Potsdam, Golm, Germany; 9 School of Life Sciences, University of Warwick, Coventry, United Kingdom; 10 Department of Aquatic Microbiology, University of Amsterdam, Amsterdam, The Netherlands; Belgian Nuclear Research Centre SCK/CEN, Belgium

## Abstract

*Nodularia spumigena* is a filamentous diazotrophic cyanobacterium that dominates the annual late summer cyanobacterial blooms in the Baltic Sea. But *N. spumigena* also is common in brackish water bodies worldwide, suggesting special adaptation allowing it to thrive at moderate salinities. A draft genome analysis of *N. spumigena* sp. CCY9414 yielded a single scaffold of 5,462,271 nucleotides in length on which genes for 5,294 proteins were annotated. A subsequent strand-specific transcriptome analysis identified more than 6,000 putative transcriptional start sites (TSS). Orphan TSSs located in intergenic regions led us to predict 764 non-coding RNAs, among them 70 copies of a possible retrotransposon and several potential RNA regulators, some of which are also present in other N2-fixing cyanobacteria. Approximately 4% of the total coding capacity is devoted to the production of secondary metabolites, among them the potent hepatotoxin nodularin, the linear spumigin and the cyclic nodulapeptin. The transcriptional complexity associated with genes involved in nitrogen fixation and heterocyst differentiation is considerably smaller compared to other Nostocales. In contrast, sophisticated systems exist for the uptake and assimilation of iron and phosphorus compounds, for the synthesis of compatible solutes, and for the formation of gas vesicles, required for the active control of buoyancy. Hence, the annotation and interpretation of this sequence provides a vast array of clues into the genomic underpinnings of the physiology of this cyanobacterium and indicates in particular a competitive edge of *N. spumigena* in nutrient-limited brackish water ecosystems.

## Introduction

Toxic cyanobacterial blooms in aquatic ecosystems are a world-wide problem, which are predicted to increase according to the present scenarios of climate change [1]. Here, we report the results of a draft genome analysis targeting *Nodularia spumigena* sp. CCY9414 (from here on *N. spumigena* CCY9414), a toxin-producing, N2-fixing, filamentous cyanobacterium isolated from the brackish waters of the southern Baltic Sea. *N. spumigena* as member of the Nostocales has a complex lifestyle, capable of cell differentiation within their long trichomes [2]. This cyanobacterium can differentiate vegetative cells into akinetes, heterocysts or hormogonia. Heterocysts are specialized cells for N2-fixation, which develop a thick cell wall and have lost photosystem II in order to decrease the internal oxygen concentration to a level that allows nitrogenase activity during the day time (for reviews see [3], [4]). Heterocysts are usually only formed when combined nitrogen is not available, but in *N. spumigena* AV1 heterocyst differentiation appeared to be uncoupled from the nitrogen supply [5]. Akinetes are cell types that serve the long-term survival of the organism under stress and non-growth permitting conditions. It is thought that *N. spumigena* forms akinetes in the Baltic Sea during autumn. The akinetes sink and overwinter in the bottom sediments from where they may be mixed back into the water column during spring and as such serve as the inoculum for a new population [6]. Hormogonia are short motile trichomes consisting of small-sized vegetative cells. They are formed from akinetes or from vegetative cells and serve the dispersal of the organism.

**Figure 1:**
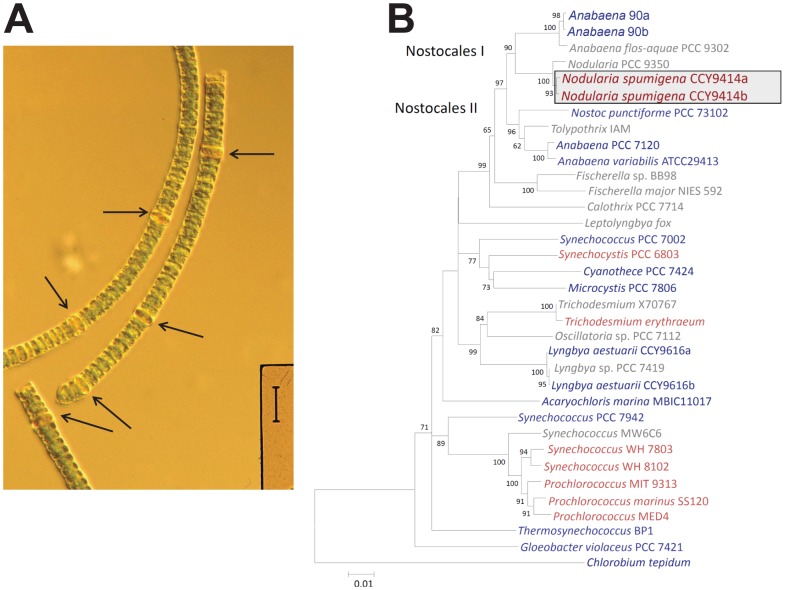
General features of *N. spumigena* CCY9414. **A**. Photomicrograph of *N. spumigena* CCY9414 trichomes. The arrows point to heterocysts. The vertical bar corresponds to 40 µm. **B**. Phylogenetic position of *N. spumigena* CCY9414 (boxed) within the cyanobacterial phyum, based on its two 16S rRNA sequences (labeled a and b). The two sub-clades within the Nostocales, clade I and clade II, are indicated. Species for which a total genome sequence is publicly available, are in blue. The sequence of *Chlorobium tepidum* TLS served as outgroup. The numbers at nodes refer to bootstrap support values (1000 repetitions) if >60%. The phylogenetic tree was generated using the Minimum Evolution method within MEGA5 [158]. The optimal tree with the sum of branch length  =  0.85445647 is shown. The tree is drawn to scale, with branch lengths in the same units as those of the evolutionary distances used to infer the phylogenetic tree and are given in the number of base substitutions per site. The multiple sequence alignment was shortened to a total of 1407 positions in the final dataset to include also 16S rRNA sequences from species without a genome sequence.

Heterocystous cyanobacteria of the group Nostocales can be divided into two major groups. There are several genome sequences available for the clade encompassing species such as Nostoc *punctiforme* ATCC 29133, *Anabaena* sp. PCC 7120 (from here *Anabaena* PCC 7120) and Anabaena *variabilis* ATCC 29413, whereas for the other clade, including *Nodularia* (**Fig. 1**), genome-level studies have only recently been started [7]. The strain *N. spumigena* CCY9414 was isolated from brackish surface waters of the Baltic Sea (near Bornholm). This isolate is a typical representative of the bloom-forming planktonic filamentous N2-fixing cyanobacteria and an important component in an ecological context. These cyanobacteria release considerable amounts of the ‘new’ nitrogen fixed into the nitrogen-poor surface waters, thereby feeding the rest of the community with a key nutrient. They contribute an estimated annual nitrogen input almost as large as the entire riverine load and twice the atmospheric load into the Baltic Sea proper [8], [9].

However, a major concern is the toxicity of these blooms, which may severely interfere with human activities [10], [11] and regularly causes animal poisonings in coastal regions of the Baltic Sea (e.g. [12], [13]). For instance, *N. spumigena* produces the potent hepatotoxin nodularin [10] but it is still unclear to what extent the toxic blooms impact on related food chains. High phosphorus combined with low to undetectable nitrogen concentrations during the summer season (hence low N∶P ratios) are principal factors favouring growth and bloom formation of *Nodularia* in the stratified Baltic Proper and Gulf of Finland [14]. This phenomenon is particularly pronounced under periods of stably stratified warm water conditions when its gas vesicles provide buoyancy leading to the formation of large surface scums in the absence of mixing. The decomposition of such blooms causes depletion of dissolved oxygen contributing to anoxic bottom waters across large areas of the Baltic.

Thus, as a diazotroph, *N. spumigena* has a selective advantage under the virtually nitrogen-free, stably stratified warm brackish water conditions of the Baltic Sea with its salinity gradient from 28 practical salinity units (PSU, equivalent to permille) to almost freshwater conditions in the surface waters above the pycnocline. In the central Baltic Sea, the preferred habitat of *N. spumigena*, the salinity varies between 1*–*2 PSU. *N. spumigena* is found at similar locations throughout the world, where brackish water conditions prevail, for instance in the Peel-Harvey inlet (Western Australia [15], [16]), or the Neuse River estuary (USA, [17]). In Australian brackish waters, *N. spumigena* blooms usually form between spring and autumn. The primary motivation for this study was to obtain genomic information from brackish-water-adapted, bloom-forming and toxic cyanobacteria, in order to gain insights into adaptations permitting it to dominate in brackish water environments. The draft genome sequence of *N. spumigena* CCY9414 allows a comparative genome analysis of its physiological capabilities. The genome analysis was complemented by a transcriptome-wide mapping of transcriptional start sites (TSS) to be able to set its regulatory complexity in the context of previously studied cyanobacteria *Synechocystis* 6803 and *Anabaena* 7120 [18], [19] and to identify the suite of putative non-coding RNAs (ncRNAs) [20], [21].

## Results and Discussion

### General Genomic Properties

The 16S rRNA-based phylogenetic tree of cyanobacteria shows two clades containing representatives from the Nostocales, clade I and clade II (**Fig. 1**). *N. spumigena* CCY9414 is located in clade I as opposed to clade II containing the much better studied Nostocales *Anabaena* PCC 7120 and *N. punctiforme* ATCC 29133 (PCC 73102). As in the closely related *Anabaena* sp. 90 [7], and some other related cyanobacteria [22], there are two 16S rRNA genes, which differ by 4 nt (99% identity), labelled *Nodularia* CCY9414 a and b. These 16S rRNA genes are associated with two distinct ribosomal RNA operons characterized by their different intergenic transcribed spacer types, one also containing the tRNA-IleGAT and tRNA-AlaTGC genes, whereas the other is lacking these tRNA genes, as also previously described for the section V cyanobacterium Fischerella sp. RV14 [22].

As summarized in Table 1, the *N. spumigena* CCY9414 draft genome sequence is distributed over 264 contigs. From these, one major scaffold of 5,462,271 nt length and several short scaffolds (<2 kb) were assembled. With this length, the genome appears smaller than those of several other Nostocales sequenced before (6.34, 6.41, 7.75 and 8.23 Mb for *Anabaena variabilis* ATCC 29413, *Anabaena* 7120, Trichodesmium erythraeum IMS101 and Nostoc *punctiforme* PCC 73102) but larger than the minimal Nostocales genomes of Cylindrospermopsis raciborskii CS-505 and Raphidiopsis brookii D9 (3.9 and 3.2 Mb [23]) and is comparable to the genome of *Anabaena* sp. strain 90 [7]. The genomic GC content is 42% and 5,294 protein-coding genes were modeled. We predicted 48 tRNA genes and one tmRNA gene. The tRNA-Leu^UAA^ gene contains a group I intron, which has been suggested as being of ancient origin [24], while the gene for the initiator tRNA, tRNA-fMet^CAT^, is intron-free, different from its ortholog in some other cyanobacteria [25].

**Table 1:** General genome and annotation information.

	*N. spumigena* CCY9414
Genome Length	5,462,271
Scaffolds	1
Avg. Contig Size	26,308
Genomic GC%	42.00%
Genes	5,294
Coding%	80.00%
tRNA Count	48
rRNA Count	8
Introns	1
sRNAs	4
Inteins	2
Sigma factors	8
Total number of TSS	6,519
Number of gTSS	1,628
Number of aTSS	2,084
Number of iTSS	2,043
Number of nTSS	764

For information on non-ubiquitious sRNAs, see Table 3.

The annotated scaffold of 5,462,271 nt length is available under GenBank accession number AOFE00000000, additionally the file containing all information on mapped transcriptional start points (TSS) can be downloaded from http://www.cyanolab.de/suppdata/Nodularia_genome/Nodularia_spumigena_CCY9414.gbk.

### Mobile Genetic Elements

*N. spumigena* CCY9414 possesses 164 genes encoding transposases. These transposases were identified by BLASTp searches against the ISfinder [26] requiring a BLASTp value of ≤1e10^−5^ and were assigned to 11 different families, each containing 1*–*32 identical copies, with highest copy numbers found for the IS200/IS605, IS607 and IS630 families of IS elements (Table S1). The large number, the high sequence similarity and the fact that active promoters were detected for many of the transposases indicate that a large part of the mobile genetic elements associated with them are active. Nevertheless, when normalized to the genome size, the number of transposase genes is similar to many other cyanobacteria, for instance, 70 transposase genes are present in *Synechocystis* PCC 6803 with 3.7 Mb genome size. However, *N. spumigena* CCY9414 has far fewer transposases than other marine N_2_-fixing cyanobacteria such as *Crocosphaera* sp. WH8501, which has as many as 1,211 transposase genes [27].

Another class of mobile elements in the *N. spumigena* CCY9414 genome is represented by at least two different Diversity Generating Retroelements (DGR1 and DGR2). DGRs introduce vast amounts of sequence diversity into their target genes [28], using a distinct type of reverse transcriptase (genes *nsp38130* for DGR1 and *nsp*13150 for DGR2; 70% amino acid identity). The very strong nTSS located 199 nt downstream of *nsp38130* may give rise to the ncRNA intermediate, which, following reverse transcription, is essential for homologous recombination into the target site for codon rewriting and protein diversification [28]. Following previously established protocols [29], we identified *nsp38150*, encoding a FGE-sulfatase superfamily-domain containing protein, as the likely target of DGR1. Closely related DGR systems, including homologs of the Nsp*38130* reverse-transcriptase and *Nsp38150* FGE-sulfatase superfamily proteins, exist in *N. punctiforme* PCC 73102 (Npun_F4892, Npun_F4890, Npun_F4889) and, in *Anabaena* PCC 7120 (Alr3497, Alr3495). However, the *N. spumigena* CCY9414 genome contains 70 copies(≥98% sequence identity) of this potential DGR1 ncRNA element consisting of the transcribed region, suggesting that DGR1 is a highly active retroelement that also inserts into non-coding regions independently of its codon rewriting capability.

Moreover, a free-standing *rvt* domain containing reverse transcriptase (*nsp10420*) was annotated, which belongs to the RNA-directed DNA polymerase:HNH endonuclease type. Such *rvt* domain proteins are not components of retrotransposons or viruses. These genes occur frequently in syntenic regions, evolve under purifying selection and are found in all major taxonomic groups including bacteria, protists, fungi, animals and plants, but their function is unknown [30]. These genes also exist in many other cyanobacterial genomes, exemplified by Alr7241 in *Anabaena* PCC 7120 and three paralogs in *Anabaena* sp. 90. A third type of putative reverse transcriptase is encoded by *nsp37000*.

**Figure 2:**
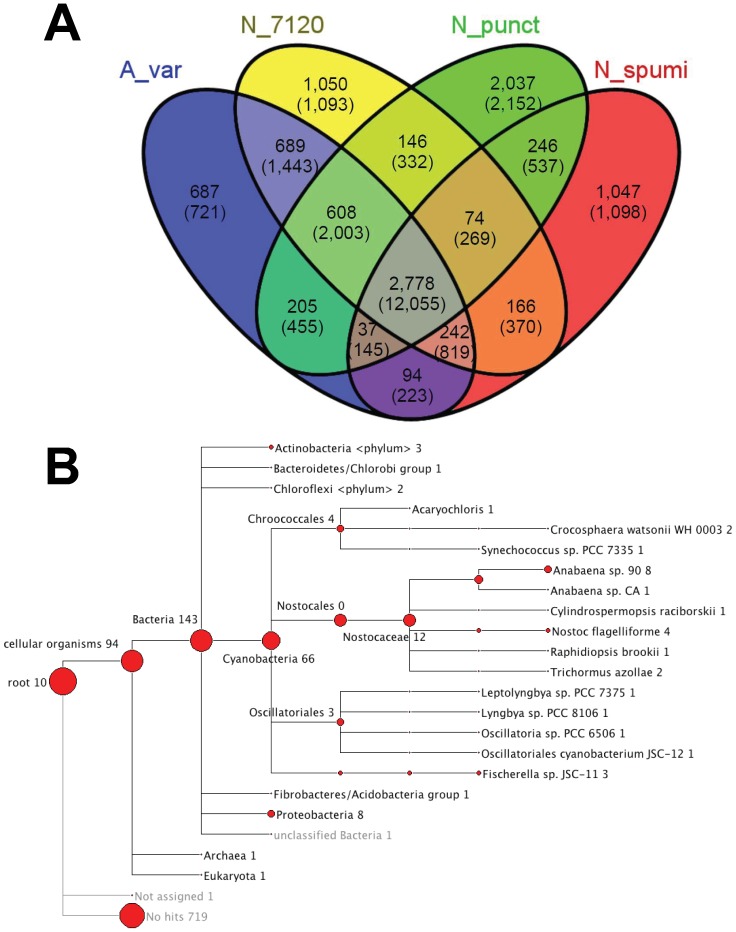
Classification of the predicted *N. spumigena* CCY9414 proteome. **A.** Comparison of all predicted proteins of *N. spumigena* (N_spumi) against the proteomes of other well-studied Nostocales, *Nostoc punctiforme* sp. PCC 73102 (N_punct), *Anabaena variabilis* sp. ATCC 29413 (A_var) and *Anabaena* PCC 7120 (N_7120) based on MCL clustering of BLASTp results (minimum e-value: 10^-8^). The numbers refer to the number of protein clusters in each category, the numbers in brackets to the total number of individual proteins. **B.** Taxonomic top hits for the 1,098 *N. spumigena* CCY9414 singletons from part A (Table S3) visualized by MEGAN.

**Fig. 2A** shows a comparison of the predicted proteome of *N. spumigena* CCY9414 with those of other well-studied Nostocales, *Nostoc punctiforme* PCC 73102, *Anabaena variabilis* ATCC 29413 and *Anabaena* PCC 7120. The core set of proteins comprises 2,778 gene clusters common to all four strains. A subgroup of these gene clusters represents multi-copy gene families of functional relevance. For example, *N. spumigena* CCY9414 harbors four identical copies of the *psbA* gene encoding the D1 protein of photosystem II, 9 copies of genes encoding proteins of the CAB/ELIP/HLIP superfamily but 2 *hetP*-like genes proposed to be involved in heterocyst differentiation [31], whereas *Anabaena* PCC 7120 possesses 5 D1- and 8 CAB/ELIP/HLIP-coding genes but 4 different *hetP*-like genes.

There are 608 gene clusters common to the three other Nostocales with the exclusion of *N. spumigena* CCY9414 (Table S2). These are likely genes specific for the Nostocales clade II. However, with 1,098 potentially unique coding sequences (1,047 gene clusters) there are also a substantial number of proteins in *N. spumigena* CCY9414 for which no homologs exists in the clade II genomes or only at low similarity (**Fig. 2A**; Table S3). F**ig. 2B** shows the taxonomic relationships of these *N. spumigena* CCY9414 genes. The largest fraction (719 genes) could not be assigned to any phylogenetic group (i.e. have not been reported before in any other organism). About 30% of the remaining 379 genes have a clear cyanobacterial origin. Another quite large group of genes were assigned to the taxon bacteria because they could not be unambiguously assigned to a particular group.

Among the 1,098 potentially unique *N. spumigena* CCY9414 genes are genes that might be expected to be more mobile, such as several restriction-modification cassettes, glycosyltransfeases (e.g. the three genes *nsp13820–13840*), but also many genes with a surprising annotation or taxonomic relation. Noteworthy are the genes *nsp5280, nsp5300* and *nsp5310*, which resemble the genes MXAN3885*–*3883 of *Myxococcus xanthus* DK1622 for fimbrial biogenesis outer membrane proteins functional in spore coat biogenesis [32].

In accordance with the planktonic lifestyle of *N. spumigena* CCY9414, ten genes *gvpA1A2CNJKLFGVW* (*nsp15380- nsp15470*) for gas vesicle proteins are arranged in one consecutive stretch of 6,372 nt that are critical for the regulation of buoyancy and are not found in benthic *N. spumigena* [33], [34].

### Organization of the Primary Transcriptome

The draft genome sequencing of *N. spumigena* CCY9414 was combined with an analysis of its transcriptome. Following established approaches for a transcriptome-wide mapping of TSS [18], [19], we analyzed a cDNA population enriched for primary transcripts obtained from an RNA sample of *N. spumigena* CCY9414 grown under standard conditions. In total, 41,519,905 sequence reads were obtained, from these 40,577,305 unique reads were mapped to the *N. spumigena* CCY9914 scaffold. The majority of these, 28,214,827 (70%) unique reads, amounting to 2,819,120,699 bases of cDNA, represented non-rRNA sequences, indicating a very high efficiency of the rRNA depletion and cDNA preparation. Applying a minimum threshold of 280 reads originating within a 7-nt window, 6,519 putative TSS were identified. In the absence of information about the real lengths of 5′ UTRs, all TSS were classified based on their position and according to published criteria [19]. Hence, all TSS within a distance of ≤200 nt upstream of an annotated gene were categorized as gene TSS. TSSs within a protein-coding region, which frequently also contribute to the generation of mRNAs, were classified as internal TSS (iTSS). TSSs for non-coding RNAs were found on the reverse complementary strand for antisense RNAs (aTSS) or within intergenic regions for non-coding sRNAs (nTSS) (Table 1). According to this classification, only 25% (1,628 gTSS) of all TSS were in the classical arrangement 5′ of an annotated gene. However, similar observations have been made during genome-wide TSS mapping in other bacteria, including the cyanobacteria Synechocystis 6803 [18] and *Anabaena* 7120 [19]. The TSS associated with the by far highest number of reads is located upstream of one of the psbA genes (psbA1, *nsp5370*). The 50 gTSS associated with the highest numbers of reads (Table 2) comprise one additional member of the psbA gene family (*psbA4*, *nsp35290*), together with seven further photosynthesis-related genes (*psbV*, *cpcG3*, transport proteins for inorganic carbon and carbon concentration and Calvin Cycle proteins). One of the genes in this category encodes a CP12 protein (Table 2). CP12 proteins are small regulators of the Calvin cycle in response to changes in light availability, but recent evidence suggests additional functions of CP12 proteins in cyanobacteria [35]. A functional class of similar size within this top-50 group of gTSS drives the transcription of translation-related genes for ribosomal proteins (S14, S16, L19, L32 and L35), the DnaJ chaperone, or translation factor IF3. The fact that photosynthesis- and translation-related gTSS are so dominant in the top-50 group illustrates that photosynthetic energy metabolism and protein biosynthesis were highly active in the culture taken for RNA analysis.

**Table 2:** The 50 gTSS of protein-coding genes associated with the highest number of reads.

Position	S	Reads	ID	Gene	Annotation
512315	+	4058097	*nsp5370*	*psbA1*	photosystem II protein D1
1911567	+	420026	*nsp19010*	*rbpA2*	RNA-binding protein
5407423	-	404779	*nsp53100*	*rbpA1*	RNA-binding protein
818076	+	254679	*nsp8200*	*rps16*	SSU ribosomal protein S16p
963628	+	194603	*nsp9560*	*-*	hypothetical protein
4901855	+	186690	*nsp48610*	*rpl32*	LSU ribosomal protein L32p
3168087	+	168603	*nsp31110*	*rps14*	SSU ribosomal protein S14p (S29e)
429606	+	161747	*nsp4480*	*-*	unknown protein
2033905	+	140335	*nsp20320*	*infC*	Translation initiation factor 3, TSS2
1997768	+	135635	*nsp19930*	*sbtA*	putative sodium-dependent bicarbonate transporter
25246	+	108746	*nsp280*	*-*	hypothetical protein
125437	+	74766	*nsp1390*	*-*	unknown protein
818072	+	69294	*nsp8200*	*rps16*	SSU ribosomal protein S16p
3595360	+	67550	*nsp35290*	*psbA4*	photosystem II protein D1 (PsbA)
2100481	−	66076	*nsp20970*	*-*	hypothetical protein
490455	+	61106	*nsp5100*	*psbV*	Photosystem II protein *PsbV*, cytochrome c550
1301909	−	58894	*nsp12740*	*glyA*	Serine hydroxymethyltransferase (EC 2.1.2.1)
3327951	+	54540	*nsp32680*	*-*	DnaJ-class molecular chaperone
4211981	−	54170	*nsp41850*	*hliE*	CAB/ELIP/HLIP superfamily
4066960	+	52955	*nsp40310*	*ccmK*	Possible carbon dioxide concentrating mechanism protein CcmK
3743395	−	51014	*nsp36850*	*-*	Branched-chain amino acid permeases, DUF4079
2477247	+	48670	*nsp24980*	*cpcG3*	Phycobilisome rod-core linker polypeptide, phycocyanin-associated
2126456	+	46218	*nsp21270*	*acpP*	Acyl carrier protein
3427650	−	45498	*nsp33690*	*-*	hypothetical protein
4477663	−	45304	*nsp44370*	*purS*	Phosphoribosylformylglycinamidine synthase, PurS subunit (EC 6.3.5.3)
4875128	−	44715	*nsp48350*	*prx5*	Peroxiredoxin
3279390	+	43459	*nsp32180*	*chlL*	Light-independent protochlorophyllide reductase iron-sulfur ATP-binding protein ChlL
308174	−	39899	*nsp3210*	*-*	hypothetical protein
4424601	+	36350	*nsp43850*	*ndhN*	Putative subunit N of NAD(P)H:quinone oxidoreductase
3660323	−-	35393	*nsp35880*	*-*	Peptidase M23B precursor
2945315	−	34180	*nsp28800*	*-*	FIG00871618: hypothetical protein
2963323	+	33480	*nsp29010*	*trxA*	Thioredoxin
2609225	−	33225	*nsp26260*	*rbcL*	Ribulose bisphosphate carboxylase large chain (EC 4.1.1.39)
948959	−	32821	*nsp9420*	*rpl19*	LSU ribosomal protein L19p
2961668	−	32694	*nsp28990*	*-*	Transposase, OrfB family
1827328	+	32133	*nsp18160*	*fbpI*	Fructose-1,6-bisphosphatase, GlpX type (EC 3.1.3.11)/Sedoheptulose-1,7-bisphosphatase (EC 3.1.3.37)
1563165	−	32001	*nsp15230*	*rpaC*	putative regulator of phycobilisome association C
4656026	−	31933	*nsp46210*	*chlP*	Geranylgeranyl reductase (EC 1.3.1.83)
1811000	+	30834	*nsp17890*	*rpoZ, ycf61*	DNA-directed RNA polymerase omega subunit
4154749	−	30790	*nsp41280*	*-*	hypothetical protein
2033866	+	29710	*nsp20320*	*infC*	Translation initiation factor 3, TSS1
2898416	+	29643	*nsp28400*	*-*	hypothetical protein
3831169	−	29380	*nsp37800*	*-*	hypothetical protein
4611031	+	29275	*nsp45760*	*rpl35*	LSU ribosomal protein L35p
175701	+	29188	*nsp1870*	*-*	hypothetical protein
4907404	+	29084	*nsp48690*	*-*	Protein CP12, regulation of Calvin cycle
4319669	-	29012	*nsp42500*	*-*	hypothetical protein
3648306	−	28409	*nsp35770*	*metX*	S-adenosylmethionine synthetase (EC 2.5.1.6)
472034	+	27553	*nsp4910*	*ubiB*	Ubiquinone biosynthesis monooxygenase UbiB
3264182	−	25978	*nsp31980*	*-*	hypothetical protein

For each gTSS, the position with respect to the forward strand, the orientation (S), the number of reads, the gene ID, gene name (if known) and gene annotation is given. The gTSS are ordered according to the number of reads.

Some of the mapped TSS gave rise to orthologs of non-coding transcripts in other cyanobacteria. For instance, the *Anabaena* PCC 7120 gene all3278, whose mutation leads to the inability to fix N2 in the presence of O2 [36], was associated with an asRNA [19]. This was also observed for the *N. spumigena* CCY9914 homolog *nsp15990*. Another example is the conservation of the nitrogen-stress-induced RNA 3 (NsiR3) first observed in *Anabaena* PCC 7120. NsiR3 is a 115 nt sRNA that is strongly induced upon removal of ammonia and controlled by an NtcA binding site [19]. The homolog in *N. spumigena* CCY9914 is transcribed from an nTSS at position 2888943, structurally conserved and also associated with a putative NtcA binding site (GTG-N8-TAC) centered at position -41. An overview of identified ncRNAs and further details are presented in Table 3.

**Table 3:** Selected non-coding RNA elements mentioned in the text or ubiquitious among bacteria.

Gene	Product	From	To	S	Comment	Reference
*ssrA*	tmRNA	3524428	3524830	+		[159], [160]
*ssaA*	6S RNA	3750385	3750114	-	single TSS located in *purK* (*nsp26940*) gene, two TSS identified in other cyanobacteria	[161]
*Ffs*	scRNA, 10S RNA	2912155	2912257	+		
*rnpB*	RNAse P RNA subunit	5025615	5025994	+		
*yfr2*	Yfr2	460195	460090	-		[162]
*nsiR3*	NsiR3	2888944	2888814	-	NtcA binding site conserved	[19]

The respective gene name is given together with the sRNA product, the location, orientation (S; +, forward strand; -, reverse strand), comments and references.

The transcriptome analysis allowed insight into the expression and promoter organization of genes involved in highly divergent physiological processes. This information is available by downloading the annotated genbank file associated with this manuscript under http://www.cyanolab.de/suppdata/Nodularia_genome/Nodularia_spumigena_CCY9414.gbk. In the following, we analyzed in more detail genes involved in the formation of heterocysts, the regulation of nitrogen metabolism and N2 fixation that were transcribed from highly active TSS. The global nitrogen regulatory protein NtcA was transcribed from a single TSS located 45 nt upstream of the start codon, associated with a perfect (GTA-N8-TAC) NtcA-binding motif [37]. In comparison, six different TSS were reported for the ntcA gene in *Anabaena* PCC 7120 [19], [38], [39]. Similarly, *N. spumigena* CCY9414 *hetR* was transcribed from a single TSS 109 nt upstream of the start codon, compared to four TSS driving the transcription of *hetR* in *Anabaena* PCC 7120 [19], [38], [40], [41]. It should be stressed that the multiple TSS in *Anabaena* PCC 7120 were detected by the same approach in the absence of combined nitrogen [19] as used here for *N. spumigena* CCY9414. Therefore, these genes that code for proteins central for the differentiation of heterocysts and N2 fixation appear to be controlled from less complex promoter regions in *N. spumigena* CCY9414 when compared to the well-studied *Anabaena* PCC 7120. A simplified genome/transcriptome arrangement was also detected for the genes encoding glutamine synthetase and the glutamine synthetase inactivating factor IF7, glnA and gifA (*nsp16180* and *nsp16190*). In *Anabaena* PCC 7120, these genes have six [19], [42]–[44] and one TSS [45] and are arranged tail-to-tail. This genomic arrangement is also conserved in *N. spumigena* CCY9414 but only three TSS were detected for glnA (Table 4). The phycobilisome degradation protein NblA is another example from this set: it has five TSS mapped by dRNAseq and confirmed by primer extension in *Anabaena* PCC 7120 [19], but only two TSS were detected upstream of the *N. spumigena* CCY9414 homolog (*nsp44910*), at positions -102 and -340. In contrast to these examples, genes not involved in nitrogen assimilation exhibited a conserved promoter architecture. For example the *rbcLXS* operon is transcribed from two TSS at positions -25 and -504 in *Anabaena* PCC 7120 [19], [46]–[48] and this is also the case in *N. spumigena* CCY9414, at positions -31 and -512.

**Table 4:** Selected proteins of heterocyst differentiation, pattern formation and nitrogen assimilation in *N. spumigena* CCY9414.

Category/Protein	*N. spumigena* CCY9414		*Anabaena* PCC 7120	
	NsORF	TSS	Identity	e-value
**Early events**				
NtcA^§^	*nsp2630*	254230R	99%	e-121
HanA, HupB	*-*	-	-	-
HetR^§^	*nsp16830*	1713443R	91%	e-162
HetF	*nsp22100*	2206991F	61%	0
HetC	*-*	-	-	-
HetL	-	-	-	-
HetP (alr2818)^§^	*nsp7850*	-	69%	2e-046
NrrA^§*^	*nsp18040*	1819958R	92%	1e-134
HetZ*	*nsp39970*	4037960F	90%	1e-173
**Pattern formation**				
PatS	*nsp47965*	-	82%	0.011
PatA*	*nsp24860*	-	61%	1e-130
PatB	*nsp41440*	-	81%	0
PatN*^§^	*nsp12530*	1277587F, 1277705F	71%	5e-084
HetN	*-*	-	-	-
**Nitrogen assimilation**				
GlnA^§*^	*nsp16180*	1656268F, 1656364F, 1656438F	88%	0
GifA	*nsp16190*	1658460R	79%	3e-025
NblA^§^	*nsp44910*	4524908F	92%	1e-029

The protein names are given, followed by the ORF ID in *N. spumigena* CCY9414 (NsORF), the position of the TSS (F, forward or R, reverse strand), the% ID and e-value in a pairwise alignment with the orthologs from *Anabaena* PCC 7120. Only amino acid identities ≥60% were considered; (-) not detected; *gene is associated with an antisense RNA in in *N. spumigena* CCY9414; ^§^gene is associated with multiple TSS in *Anabaena* PCC 7120.

### Genome-Based Prediction of Compatible Solute Accumulation Capabilities

Analysis of salt-induced compatible solute accumulation in approximately 200 different cyanobacterial strains proposed that freshwater and brackish water strains (low salt resistance) accumulate the disaccharides sucrose and/or trehalose, while true marine strains (moderate salt tolerance) contain the heteroside glucosylglycerol (GG), and halophilic and hypersaline strains accumulate betaines, mainly glycine betaine (reviewed in [49]). Salt-loaded cells of Nostocales accumulated only disaccharides, in agreement with their low salt tolerance. *N. spumigena* CCY9414 occurs in the Baltic Sea mainly at salinities ranging from 2 – 10 PSU (equivalent to 0.2 – 1% NaCl and 5 – 33% of full seawater salinity). Its genome was searched using sucrose-phosphate synthase (SpsA) and sucrose-phosphate phosphatase (Spp) sequences from *Synechocystis* sp. PCC 6803 (*sps*–*sll0045*, *spp*–*slr0953* [50]). The *N. spumigena* CCY9414 ORF *nsp8740* shows significant similarities to SpsA, which clusters with similar enzymes from unicellular cyanobacteria, while putative Sps proteins from Nostocales were found in a separate clade (**Fig. S1**). The *nsp8740* gene was found to be highly expressed under our standard cultivation conditions (10 PSU) explaining the observed sucrose accumulation in these cells (F. Möke, unpublished). The Sps of *N. spumigena* CCY9414 could be a combined enzyme with Sps as well as Spp activity, because both domains are present in the sequence of ORF *nsp8740*. An apparently truncated Sps protein is encoded by *nsp23670*, with closely related proteins in other Nostocales (**Fig. S1**). Similar to Synechocystis sp. PCC 6803 and *Anabaena* PCC 7120, the *N. spumigena* CCY9414 genome also harbors a single spp gene (*nsp21420*). *Anabaena* PCC 7120 also possesses sucrose synthase (susA - all4985, susB – all1059) [51]. Similar proteins, SusA (*nsp24720*) and susB (*nsp48150*) were also found. Hence, sucrose metabolism in *N. spumigena* CCY9414 is similar to *Anabaena* PCC 7120, where Sps is used to synthesize sucrose to serve as a compatible solute as well as serving as a source of energy and reducing power for N2-fixation in the heterocyst [52]: SusA and SusB are probably involved in sucrose breakdown to provide electrons and energy for N_2_-fixation [51], [53].

Low salt tolerant cyanobacteria often also accumulate trehalose, which is usually synthesized by the maltooligosyl trehalose synthases (*Mts1* and *Mts2*) using glycogen as precursor. This trehalose synthesis pathway often includes also TreS as enzyme capable of hydrolyzing trehalose into glucose (e.g. [54]). In *Anabaena* PCC 7120, an operon was identified comprising the *treS*, *mts1* and *mts2* genes (*all0166, all0167, all0168*; [55]). Proteins very similar to *Mts1* and *Mts2* are encoded in *N. spumigena* CCY9414 by two genes likely forming an operon (*nsp*41870*/nsp41880*). These genes are linked to *nsp41890* (the first gene in a putative operon with *mts1* and *mts2*) that encodes for a glycogen de-branching enzyme, making the precursor glycogen for trehalose synthesis accessible. Finally, ORF *nsp39450* is a good candidate encoding TreS for degradation of trehalose. Hitherto, there is no experimental verification for trehalose accumulation in *N. spumigena* CCY9414, i.e. cells grown in liquid media at different salinities accumulated only sucrose (F. Möke, unpublished). The absence of trehalose correlates well with the absence of an active promoter for the *mts1*/*2* genes under the growth conditions tested here. In this respect, it is interesting to note that salt-stressed cells of *Anabaena* PCC 7120 also only accumulate sucrose ([52]; own observations), while the trehalose biosynthesis genes were induced upon desiccation in this organism [56], [57].

Besides *de novo* synthesis, compatible solutes are often sequestered via specific transporters. *N. spumigena* CCY9414 contains multiple genes for such transporters. An ABC-type transporter for glycine betaine/choline uptake [58], [59] appears to be encoded by *nsp43160* to *nsp43200*. Another gene cluster seems to encode a proline/glycine betaine ABC transporter (*nsp6940/nsp6950*). In contrast, an ABC-type transporter for compatible solutes sucrose, trehalose, and GG, such as GgtABCD from *Synechocystis* sp. PCC 6803 [49], was not found in the *N. spumigena* CCY9414 genome. The presence of multiple compatible solute uptake systems might be favorable in complex microbial communities, in which dissolved compatible solutes such as proline and glycine betaine released from other microbes can be quickly taken up and used in addition to the *de novo* biosynthesis of sucrose.

### Acclimation Strategies to Low Iron Levels: a Multitude of Transport Systems

Iron is one of the main factors determining cyanobacterial productivity in the marine pelagic environment including cyanobacterial blooms in the Baltic Sea [60], because most inorganic iron in the oxygenated biosphere was converted into virtually insoluble ferric iron. Acclimation of cyanobacteria to iron starvation includes the induction of specific transport systems [61]. *Synechocystis* sp. PCC 6803 possesses at least three ABC-type iron-specific transporters, which seem to be specialized for uptake of Fe^2+^ (*feoB*, *slr1392,* etc.), Fe^3+^ (*futA*, *slr1295/slr0513*, etc.), and Fe^3+^-dicitrate (*fecB, sll1202 or slr1491*, etc.). Similar gene clusters are present in the genome of *Anabaena* PCC 7120, which encodes multiple copies of the fec operon [62]. These genes were used to search the genome of *N. spumigena* CCY9414 (Table 5). Corresponding to the ecological niche, the genome of *N. spumigena* CCY9414 lacks a Fe^2+^ uptake system of the Feo-type, which is consistent with the nearly complete absence of Fe^2+^ in the oxygenated seawater environment of *N. spumigena*. However, as expected for an organism that is exposed to iron limitation, at least three alternative iron uptake systems were found. One operon contains four genes similar to the fut operon (*nsp19100-nsp19130*), which encode an ABC-type Fe^3+^ uptake system. Additionally, two systems for the uptake of Fe^3+^ bound to organic chelators (siderophores), such as dicitrate or hydroxamate exist in *N. spumigena* CCY9414. One of these transporters is similar to the Fec system from *Synechocystis* sp. PCC 6803 or *Anabaena* PCC 7120 (*fecBEDC*; *nsp11930-nsp11960*). It is linked to a TonB-dependent ferrichrome-like receptor (*nsp11910*) used for the uptake of chelated Fe^3+^ [62]. This protein also shows similarities to Alr0397, which was characterized as the receptor for the siderophore schizokinen in *Anabaena* PCC 7120 [63]. The genes putatively involved in schizokinen synthesis in *Anabaena* PCC 7120 [63] were not found in the genome of *N. spumigena* CCY9414. However, the *N. spumigena* CCY9414 genome contains an *fhuCDB* operon (*nsp27490-nsp27510*), annotated as a Ferric-hydroxamate ABC transporter. This implies that *N. spumigena* CCY9414 is able to accept many forms of chelated Fe^3+^ including those bound to siderophores produced by other bacteria present in the brackish water community.

**Table 5:** Proteins related to the uptake of iron in *N. spumigena* CCY9414 identified on the basis of gene clusters present in the genome of *Anabaena* PCC 7120.

ORF	NsORF	Annotation	% ID	Reference
**Operon I**				
*All2618*	*nsp11930, nsp2720*	Iron(III) dicitrate transport system, periplasmatic Iron(III) dicitrate transport system, periplasmatic	36, 32	
*All2619* Ferrobactin Receptor	*nsp11910, nsp2710*	TonB-dependent receptor; Outer membrane Ferrichrome receptor	50, 25	
*All2620,* Ferrobactin Receptor	*nsp11910, nsp2710, nsp26750*	TonB-dependent receptor; Outer membrane Ferrichrome-iron receptor, Ferrichrome-iron receptor	47, 38, 29	
**Operon II**				
*Alr2209* Aerobactin receptor	*nsp11910, nsp2710, nsp26750*	TonB-dependent receptor; Outer membrane Ferrichrome-iron receptor, Ferrichrome-iron receptor	46, 27, 25	
*Alr2210*	*nsp2720, nsp11930*	iron(III) dicitrate-binding periplasmic protein	38, 38	
*Alr2211* ferrichrome-iron receptor	*nsp2710, nsp26750*	Ferrichrome-iron receptor	45, 38	
*Alr2212*	*nsp11930, nsp2720, nsp27500*	Iron(III) dicitrate transport system, periplasmic proteins, Ferric hydroxamate ABC transporter	37, 32, 37	
*Alr2213*	*nsp11930 nsp2720*	Iron(III) dicitrate transport system, periplasmic proteins		
**Operon III**				
*Alr0397,* schizokinen receptor	*nsp11910, nsp2710, nsp26750*	TonB-dependent receptor; Outer membrane, Ferrichrome-iron receptor, Ferrichrome-iron receptor	47, 37, 24	[164]
*All0396*, iaminobutyrate--pyruvate transaminase	*nsp46650*	Acetylornithine aminotransferase	29	[164]
*All0395,* L-2,4-diaminobutyrate decarboxylase	*nsp37340*	Cysteine desulfurase	26	[164]
**Operon IV**				
*All0390, rhbF*	*nothing*			[62]
*All0389, fhuC*	*nsp27490, nsp11960*	Ferric hydroxamate ABC transporter, ABC-type Fe3+-siderophore transport system, ATP-binding	89, 50	[62]
*All0388, fhuD*	*nsp27500 nsp11930*	Ferric hydroxamate ABC transporter Iron(III) dicitrate transport system, periplasmic binding	83,30	[62]
*All0387, fhuB*	*nsp27510, nsp11950, nsp11940*	Ferric hydroxamate ABC transporter, ABC-type Fe3+-siderophore transport system, permeases, Nsp11950 and 11940 probably 1 ORF	85, 37, 48	[62]
**Operon V**				
*Alr2581* aerobactin receptor	*nsp11910, nsp2710, nsp26750*	TonB-dependent receptor; Outer membrane Ferrichrome-iron receptor, Ferrichrome-iron receptor	78, 27, 24	
*Alr2582,* Hyp. Prot.	*nsp28410*	hypothetical protein	47	
*Alr2583, fecB1*	*nsp11930, nsp2720*	Iron(III) dicitrate transport system, periplasmic binding protein	80, 36	[62]
*All2584, fecE1*	*nsp11960, nsp27490*	ABC-type Fe3+-siderophore transport system, ATP-binding Ferric hydroxamate ABC transporter	71, 52	[62]
*All2585, fecD1*	*nsp11950, nsp11940, nsp27510*	ABC-type Fe3+-siderophore transport system, permeases, Ferric hydroxamate ABC transporter	67, 36, 43	[62]
*All2586, fecC1*	*nsp11950, nsp11940, nsp27510*	ABC-type Fe3+-siderophore transport system, permeases, Ferric hydroxamate ABC transporter	61, 37, 34	[62]
**Operon VI**				
*Alr2587,* Transcription factor	*nsp2700*	Transcriptional regulator, AraC family	38	
*Alr2588,* ferrichrome-Fe-receptor	*nsp2710, nsp26750, nsp11910*	Ferrichrome-iron receptor, Ferrichrome-iron receptor, TonB-dependent receptor	50, 40, 41	
*Alr2589,* Hyp. Prot.	*nsp28410*	hypothetical protein	47	
*Alr2590,* iron(III) dicitrate-binding protein	*nsp11930 nsp2720, nsp27500*	Iron(III) dicitrate transport system, periplasmic binding proteins, Ferric hydroxamate ABC transporter	28, 27, 37	
*Alr2591,* Transcription factor	*nsp2700*	Transcriptional regulator, AraC family	38	
*Alr2592,* ferrichrome-Fe receptor	*nsp2710, nsp26750, nsp11910*	Ferrichrome-iron receptor, Ferrichrome-iron receptor, TonB-dependent receptor	46, 38,26	
*Alr2593* iron(III) dicitrate-binding	*nsp11930,nsp2720*	Iron(III) dicitrate transport system, binding periplasmic proteins	38, 35	
*Alr2594,* hypothetical protein				
*Alr2595,* Transcription Factor	*nsp2700*	Transcriptional regulator, AraC family	40	
*Alr2596,* ferrichrome-Fe receptor	*nsp2710, nsp26750, nsp11910*	Ferrichrome-iron receptor, Ferrichrome-iron receptor, TonB-dependent receptor	49, 38, 27	
*Alr2597,* iron(III) dicitrate-binding	*nsp11930, nsp2720*	Iron(III) dicitrate transport system, periplasmic binding proteins	29, 26	
**Operon VII**				
*Alr3240, FecD2*	*nsp11950, nsp11940*	ABC-type Fe3+-siderophore transport system, permease proteins	42, 37	[62]
*Alr3241, FecE2*	*nsp11960, nsp27490*	ABC-type Fe3+-siderophore transport system, ATP-binding, Ferric hydroxamate ABC transporter	46, 44	[62]
*Alr3242, hutA2*	*nsp11910, nsp2710*	TonB-dependent receptor, Ferrichrome-iron receptor	25, 22	[62]
*Alr3243, fecB2*	*nsp11930*	Iron(III) dicitrate transport system, periplasmic binding protein	24	[62]
**Operon VIII**				
*Alr4030,* Hypot. Prot.	*nsp14910*	hypothetical protein	38	[62]
*Alr4031, fecB3*	*nsp11930*	Iron(III) dicitrate transport system, periplasmic binding protein	23	[62]
*Alr4032, fecD3*	*nsp11950, nsp11940, nsp27510*	ABC-type Fe3+-siderophore transport system, permease proteins, Ferric hydroxamate ABC transport	32, 28, 31	[62]
*Alr4033* *fecE3*	*nsp11960*	ABC-type Fe3 +-siderophore transport system, ATP-binding	33	[62]
**Operon IX**				
*Alr1381, prcA*	*nsp18110*	Calcium-dependent protease precursor	32	[62]
*Alr1382, futA*	*nsp19110, nsp19100*	Ferric iron-binding periplasmic proteins of ABC transporter	57, 49	[62]
*Alr1383, futB*	*nsp19120*	Ferric iron ABC transporter, permease protein	64	[62]
*Alr1384, futC*	*nsp19130*	Iron(III)-transport ATP-binding protein	60	[62]

The first row contains the ORF ID, annotation and gene name (if available) of the respective *Anabaena* protein (according to the published sequence [163] in Genbank file NC_003272.1), followed by the ORF ID in *N. spumigena* CCY9414 (NsORF), the detailed annotation, the% ID in a pairwise alignment and the reference, if available.

### Acclimation Strategies to Low Iron Levels: a Multitude of *psbC/isiA/pcb* Genes

**Figure 3:**
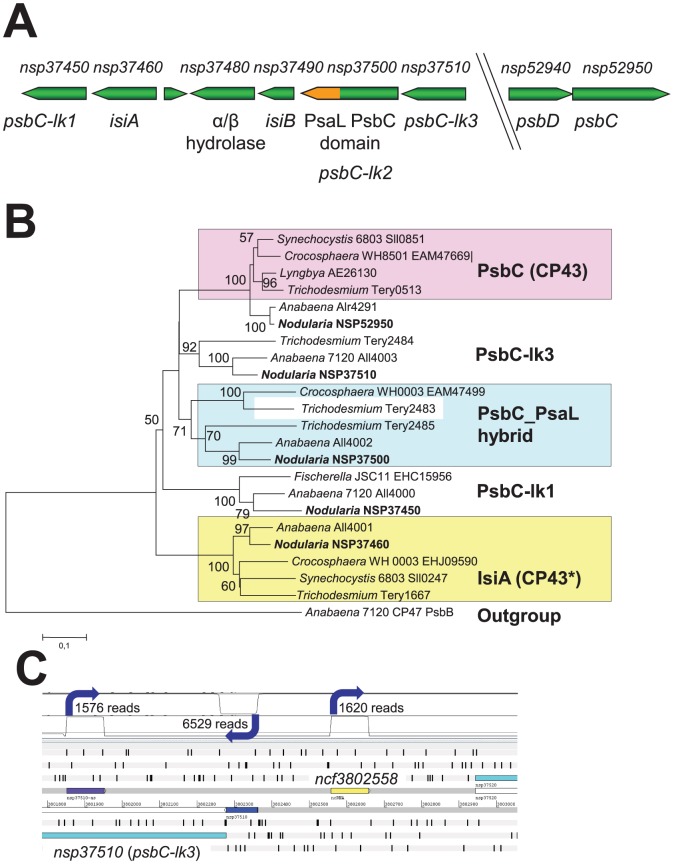
Analysis of loci encoding proteins of the CP43/IsiA/Pcb family. **A.** Organization of the chromosomal region harboring the *isiA* and *psbC*-like genes (*psbC-lk1-3*) of *N. spumigena* and the separate *psbDC* operon. The PsaL–coding domain in *psbC-lk2* (*nsp37500*) is highlighted in orange. **B.** Phylogenetic analysis of CP43, IsiA and related chlorophyll-binding proteins from *N. spumigena* and of selected other cyanobacteria was inferred using the Minimum Evolution method. The optimal tree with the sum of branch length = 3,97009738 is shown. The percentage of replicate trees in which the associated taxa clustered in the bootstrap test (1000 replicates) are shown next to the branches. The tree is drawn to scale, with branch lengths in the same units as those of the evolutionary distances used to infer the phylogenetic tree. The evolutionary distances were computed using the Poisson correction method and are in the units of the number of amino acid substitutions per site. All positions containing gaps and missing data were eliminated from the dataset (complete deletion option). There were a total of 279 positions in the final dataset. **C.** Transcriptional organization around the *isiA, isiB* and *psbC*-like gene cluster. There are three mapped TSS in the region displayed in **Fig. 3A**, all associated with or close to the 5′ end of *nsp37510.* TSS are indicated by blue arrows and the number of cDNA reads associated with them are given as approximation for their activity. One gTSS gives rise to the 83 nt long 5′ UTR upstream of *nsp37510* (blue) and the gene or operon mRNA. An antisense RNA originates from a single aTSS in the opposite direction (purple). The third TSS is a putative nTSS driving the transcription of an ncRNA in the *nsp37510*- *nsp37520* intergenic spacer. Except for the *nsp37510* 5′ UTR, all TSS displayed are drawn with a 100 nt-long box that corresponded to the maximum read length in the dRNAseq approach.

One gene that becomes strongly expressed under iron-limiting conditions in many cyanobacteria is *isiA*, coding for the iron stress induced protein A [64*]*–[66]. Additionally, IsiA participates in high light acclimation [67]. IsiA belongs, together with the CP43 (PsbC) and the Pcb's from *Prochloron*, *Prochlorothrix*, *Prochlorococcus* and *Acaryochloris,* to a family of related antenna proteins that bind chlorophylls. In *N. spumigena* CCY9414, *psbC (nsp52950)* is located at one genomic location as part of a *psbDC* dicistronic operon, which is the typical gene organization of these photosystem II core antenna genes among cyanobacteria. However, four additional genes of the *isiA/psbC/pcb* family (*nsp37450, nsp37460, nsp37500, nsp37510*) are clustered with a flavodoxin gene (*nsp37490*, *isiB*) at another site in the genome (**Fig. 3A**). In between the flavodoxin and isiA genes a protein of unknown function with an alpha/beta hydrolase domain is encoded (*nsp37480*), homologs of which are associated with flavodoxin genes also in most other N2-fixing cyanobacteria. A similar situation with several tightly clustered genes of the IsiA/CP43 family exists in *Anabaena* PCC 7120 and other filamentous, N2-fixing cyanobacteria such as Fischerella muscicola PCC 73103 [68]. A phylogenetic analysis of these proteins shows that one of the proteins from this family (*nsp37460*) clusters with several well characterized IsiA proteins and hence is a distinct IsiA homolog. In contrast, the other four proteins belong to a tight cluster also containing PsbC (**Fig. 3B**).

One of the PsbC homologs (*Nsp37500*) possesses a considerable C terminal extension (total length 477 amino acids compared to 319*–*344 residues for the other PsbC homologs). A closer inspection revealed that *Nsp37500* possesses a PsaL domain in this additional segment and that nine transmembrane regions are predicted for the PsbC-PsaL hybrid protein (SI, **Fig. S2**). Similar genes have recently been identified in several more cyanobacterial genomes and the PsbC-PsaL hybrid proteins have been classified as chlorophyll binding proteins type V (CBPV) [69]. Analysis of a PsaL-less mutant of Synechocystis sp. PCC 6803 indicated that PsaL is required for the formation PSI trimers. However, iron-starved cells of this mutant were still able to form IsiA rings around PSI monomers but to a lesser extent [66], [70]. The PsbC-PsaL fusion present in *Nsp37500* suggests that this strain is hard-wired for the addition of chlorophyll-antenna to PSI monomers over and above the IsiA-rings associated with PSI trimers. This possibility is supported by the results of a recent homology modelling and insertion of the PsaL-like domain into the PSI structure [69]. Such an antenna complex may be a particularly efficient form of light-harvesting by PSI in the ecological niche of *N. spumigena*. The regulation of these genes in *N. spumigena* is not known, but at least for *F. muscicola* PCC 73103 the iron-stress-regulation of a comparable large operon with Pcb/PsbC homologs was detected [68].

The transcriptome data provides an initial snapshot on the expression of the different members of the *psbC/isiA*-like gene family in *N. spumigena* CCY9414. While the classical *psbDC* operon is strongly expressed, we detected only a rather weak TSS associated with the genes *nsp37450, nsp37460, nsp37500* and *nsp37510*, which is located 83 nt upstream of *nsp37510* and could indicate the presence of a long operon consisting of these genes (**Fig. 3C**). However, its activity might be decreased by the activity of an aTSS at position +426 (**Fig. 3C**) under some conditions. If so, this antisense RNA could have an analogous control function as the IsrR antisense RNA to the *isiA* gene in *Synechocystis* sp. 6803 [20].

### Dinitrogen Fixation: Nitrogenase and Hydrogenases in *N. spumigena* CCY9414

*N. spumigena* CCY9414 has one complete set of *nif* genes coding for a Mo-nitrogenase 1 and additional N_2_ fixation genes within a region of 26,173 base pairs (genes *nsp40650-nsp40900*), transcribed from a single but strong TSS 296 nt upstream of *nsp40650* (*nifB*). A *nifHDK* gene cluster is present in this region, including a split *nifH* (*nsp40720*) and a split *nifD* (*nsp40770*) gene. A second copy of *nifH (nifH2; nsp34540)*, coding for dinitrogenase reductase, is present at an unrelated site in the genome. In *N. spumigena* strain AV1 expression of *nifH2* seems to be under nitrogen control [71].

*N. spumigena* CCY9414 encodes two [NiFe] hydrogenases as is the case in all other N_2_-fixing cyanobacteria investigated to date. The genes for catalytic subunits of uptake hydrogenase, *hupS* (*nsp41100*) and *hupL* (*nsp41090* and *nsp41000,* which become fused following heterocyst-specific recombination), are separated by an intergenic stretch that might form a hairpin as has been described for other cyanobacteria [72]. The genome also contains *hoxEFUYH* (genes *nsp28020-nsp28070*), encoding the structural proteins of the bidirectional hydrogenase, an enzyme common in many diazotrophic and non-diazotrophic cyanobacteria. Both uptake and bidirectional hydrogenase gene clusters possess genes for a putative endoprotease, HupW (*nsp40980*) and *HoxW* (*nsp28070*), processing the large subunits *HupL* (genes *nsp41090* and *nsp41000*) and *HoxH* (*nsp28060*), respectively. These genes are located downstream from *hupL* and *hoxH,* and in the case of the uptake hydrogenase separated from *hupL* by a small ORF. In *N. spumigena* CCY9414 the *hox* genes form a contiguous cluster *hoxEFUYHW* (*nsp28020-nsp28070*) without additional ORF's. The *hyp* genes for maturation proteins are present as single copy genes in the genome of *N. spumigena* CCY9414.

### Dinitrogen Fixation: Regulation of Heterocyst Differentiation

*N. spumigena* forms regularly spaced heterocysts along the filaments as other Nostocales. The structural genes for dinitogen fixation and heterocyst formation are closely related to those from *Anabaena* PCC 7120 (see Table 5 for overview). Regulatory proteins, such as the transcription factor NtcA (*nsp2630*), which senses the intracellular accumulation of 2-oxoglutarate as an indicator of nitrogen limitation [73] and then triggers the differentiation process towards heterocysts via *HetR* (*nsp16830*, [74]), are present in the *N. spumigena* CCY9414 genome (*nsp2630*). In many heterocystous cyanobacteria, such as *Anabaena* PCC 7120, *hetR* is expressed in a spatial pattern along the trichomes [75*]*–[78], triggered from a heterocyst-specific TSS. Many further well-characterized genes encoding protein factors involved in heterocyst formation (reviewed in [3]), such as NrrA, PatN and the signalling peptide PatS (sequence here: MKTTMLVNFLDERGSGR the minimum pentapeptide required for normal heterocyst pattern formation underlined), HetF and HetP and *hetP*-like genes (2 copies) are also present in the *N. spumigena* CCY9414 genome. HetF is required for heterocyst formation and for the normally spaced expression of *hetR* in *N. punctiforme* [79] and *Anabaena* PCC 7120 [80].

In addition to the known regulatory proteins, associated regulatory RNAs for heterocyst differentiation are well conserved in *N. spumigena* CCY9414 as they are in other Nostocales. A tandem array of 12 short repeats was found upstream of *hetF* (*nsp22100*) [81]. A homologous tandem array in *Anabaena* PCC 7120 gives rise to the NsiR1 sRNA that likely plays a role in the regulatory cascade leading to heterocyst differentiation [19], [81]. HetZ is a protein involved in *Anabaena* PCC 7120 in early heterocyst differentiation [82]. Recently, control of its transcription by a 40 nt *HetR* binding site upstream of a TSS was suggested, which is located at position -425, antisense to gene *asl0097* [83]. This arrangement is almost exactly conserved in *N. spumigena* CCY9414: the only TSS upstream of the *hetZ* homolog *nsp39970*, was mapped at position -429 and in antisense orientation to *nsp39970*, the homolog of *asl0097*. Moreover, the sequence 5′-ATTTGAGGGTCAAGCCCAGCAGGTGAACTTAGGGAGACAT-3′, located 56-17 nt upstream of this TSS is almost identical to the reported *HetR* binding site in *Anabaena* PCC 7120 [83]. These facts, together with the conserved arrangement, including a long 5′-UTR of *hetZ* and aTSS located within the gene upstream, suggest that the *HetR* binding site is also functional in *N. spumigena* CCY9414.

Genes for PatA and PatB, which play essential roles in controlling the spacing of heterocysts along a filament [84*]*–[86] are also present in the *N. spumigena* CCY9414 genome. However, other proteins involved in heterocyst formation were not found. Among these are *hetN*, in *Anabaena* PCC 7120 involved in patterning of heterocysts along the filaments, and *hanA (hupB)*, encoding the histone-like HU protein [87], which is essential for heterocyst differentiation in *Anabaena* PCC 7120 [88]. Likewise, the *hetC* gene, proposed to be expressed in pro-heterocysts and to stimulate *ftsZ* expression [89], [90], and *hetL*, which simulates heterocyst development even in the presence of combined nitrogen [91], were not found. The lack of genes for some of the proteins involved in early events of heterocyst formation indicates that *N. spumigena* CCY9414 uses a mechanism for regulating early heterocyst differentiation different from that in *Anabaena* PCC 7120. These findings correspond with the less stringent regulation of heterocyst formation by the nitrogen supply as reported for *N. spumigena* AV1 [5].

### Dinitrogen fixation: DNA Rearrangements Involved in Heterocyst Differentiation

DNA rearrangements as part of heterocyst developmental processes are known from the heterocystous cyanobacteria *Anabaena* and *Nostoc* [92], [93]. A DNA element, interrupting a gene in the vegetative cell, is excised leading to recombination and transcription of the genes in the heterocyst in order to perform the function that is heterocyst specific. In *Anabaena* PCC 7120 three DNA elements have been identified and named after the genes they interrupt: *nifD*, *fdxN* and *hupL* element. The genome of *N. spumigena* CCY9414 also is likely to undergo three DNA rearrangements; it contains a *nifD* and a *hupL,* but instead of a *fdxN* it has a *nifH1* element. However, the size of the *nifD* and *hupL* elements is smaller than in most other Nostocales. The *nifD* element of *N. spumigena* CCY9414 differs from those of other cyanobacteria also in the number of ORFs. In addition to *xisA,* which encodes the site-specific recombinase, only a single other ORF (*nsp40780*) for a hypothetical protein was identified on this element in *N. spumigena* CCY9414. The *hupL* element of *N. spumigena* CCY9414 is 7.6 kb and also smaller than the 10.5 kb element of *Anabaena* PCC 7120. Five out of 7 ORFs found on the *hupL* element in *N. spumigena* CCY9414, including the recombinase gene *xisC,* have sequence identities of 86*–*97% at the DNA level to 6 out of 10 ORFs present on the element of *Anabaena* PCC 7120. The two ORFs on the *N. spumigena* CCY9414 *hupL* element that do not have homologs on the *Anabaena* PCC 7120 element, are similar to a DNA-cytosine methyltransferase and a HNH-type endonuclease (*nsp41020* and *nsp41030*) and appear to be transcribed from a specific TSS 28 nt upstream of *nsp41020*.

The directly repeated sequences flanking the *nifD* element differ in *N. spumigena* CCY9414 by one nucleotide from each other. The repeat flanking the 5′ part of *nifD* is identical to the 11 bp sequence of other strains (GGATTACTCCG), while the repeat flanking the 3′ part of *nifD,* close to *xisA,* differs by one nucleotide (GGA**A**TACTCCG). A similar difference was observed in the element of *Anabaena* sp. ATCC33047 [94], but the differing nucleotides are not the same. Also the repeated sequences of the *hupL* element differ in *N. spumigena* CCY9414 by a single nucleotide. The repeat at the 5′ part of *hupL* is identical to the 16 bp repeat from *Anabaena* PCC 7120 (CACAGCAGTTATATGG) while the repeat close to *xisC* at the 3′ part of *hupL* is different (CATAGCAGTTATATGG). The direct repeated sequences from both *nifD* and *hupL* elements are present only once on the genome of *N. spumigena* CCY9414, thus, it appears that these excisions are very specific.

In contrast to *Anabaena* PCC 7120 no rearrangement in *fdxN* seems to take place in *N. spumigena* CCY9414. Instead a third rearrangement exists in *nifH1* in the *nifHDK* cluster. The activity of this previously unknown DNA rearrangement mechanism was recently demonstrated [71]. The *nifH1* element is 5.2 kb and encodes a XisA/XisC-type site-specific recombinase (*nsp40750*). In addition to this recombinase only two further ORFs are located on the *nifH1* element, coding for a hypothetical protein and a putative DNA modification methylase. The recombinase encoded by *nsp40750*, which we term XisG, is 48% identical to XisA and 4% identical to XisC of *N. spumigena* CCY9414. All three recombinases contain the highly conserved tetrad R-H-R-Y of the phage integrase family with the catalytically active residue tyrosine, but as was described for XisA and XisC of *Anabaena* PCC 7120 [95], the histidine is substituted by a tyrosine in *N. spumigena* CCY9414 in XisA and XisC and the newly described XisG. The identical direct repeats flanking the *nifH1* element are only 8 bp long (CCGTGAAG). These repeats are overrepresented with 111 occurrences in the genome. Therefore, how the correct direct repeats for recombination are chosen by the recombinase is an open question. Interestingly, other strains of *N. spumigena* known to develop heterocysts in the presence of combined nitrogen [5], [96], also have the *nifH1* element [71] found in *N. spumigena* CCY9414.

### Phosphate Acquisition: a Multitude of Phosphatases and Transport Systems

The importance of phosphorus as a key limiting nutrient in aquatic systems (see [97], [98]) awoke much interest in defining P-scavenging mechanisms in cyanobacteria, particularly at the genetic level (e.g. see [99*]*–[102]. This is especially relevant here since biologically available dissolved inorganic and organic phosphorus forms appear critical for *N. spumigena* bloom formation in the Baltic Sea [103], [104]. Moreover, expression of the nodularin synthetase gene cluster increases during P-depletion [105]. Based on existing information, searches of the *N. spumigena* CCY9414 genome for components of inorganic phosphate transport and assimilation were conducted (Table 6).

**Table 6:** Complement of P- and arsenate-related gene orthologs in *N. spumigena* CCY9914.

NsORF, gene name	Annotation	comment	Reference
**Inorganic P transport**			
*nsp1550*	low affinity P permease		
*nsp16870*	low affinity P permease		
*nsp15300, sphX*	freshwater *sphX* (P binding protein)	similar to *Synechocystis* PCC6803 *sll0540*	
*nsp28900, pstS1*	periplasmic P binding protein PstS	similar to *Synechocystis* PCC6803 *slr1247*	[100], [106]
*nsp28910, pstC1*	PstC component of high affinity ABC P transporter		[100], [106]
*nsp28920, pstA1*	PstA component of high affinity ABC P transporter		[100], [106]
*nsp28930, pstB1*	PstB component of high affinity ABC P transporter ATP-binding protein component		[100], [106]
*nsp28940 pstB2*	PstB component of high affinity ABC P transporter ATP-binding protein component		[100], [106]
*nsp52600, pstS2*	periplasmic P binding protein PstS	similar to *Synechocystis* PCC6803 *sll0680*	[100], [106]
*nsp52610, pstC2*	PstC component of high affinity ABC P transporter		[100], [106]
*nsp52620, pstA2*	PstA component of high affinity ABC P transporter		[100], [106]
*nsp52630, pstB2*	PstB component of high affinity ABC P transporter ATP-binding protein component		[100], [106]
**Phosphonate transport**			
*nsp7590, phnF*	PhnF component of a C-P lyase		
*nsp7580, phnG*	PhnG component of a C-P lyase		
*nsp7570, phnH2*	PhnH component of a C-P lyase		
*nsp7560, phnI*	PhnI component of a C-P lyase		
*nsp7540, phnJ*	PhnJ component of a C-P lyase		
*nsp7530, phnK*	PhnK component of a C-P lyase		
*nsp7520, phnL*	PhnL component of a C-P lyase		
*nsp7510, phnM*	PhnM component of a C-P lyase		
*nsp7490*	hypothetical protein in *phn* cluster		
*nsp7500*	hypothetical protein in *phn* cluster		
*nsp7480, phnD2*	PhnD component of phosphonate ABC transporter phosphate-binding periplasmic component		
*nsp7470, phnC2*	*PhnC* Phosphonate ABC transporter ATP-binding protein		
*nsp7460, phnE2*	PhnE Phosphonate ABC transporter permease protein		
*nsp7450, phnE3*	*PhnE3* Phosphonate ABC transporter permease protein		
*nsp35120, phnC1*	*PhnC*1 Phosphonate ABC transporter ATP-binding protein		
*nsp35130, phnD1*	PhnD1 Phosphonate ABC transporter phosphate-binding periplasmic component		
*nsp35140, phnE1*	PhnE1 Phosphonate ABC transporter permease protein		
*nsp35150, phnH1*	PhnH	truncated version, translationally coupled to *nsp35160* – PhnM component of a C-P lyase	
*nsp18360, phnD2*	PhnD2 Phosphonate ABC transporter phosphate-binding periplasmic component		
*nsp18370, phnC2*	*PhnC*2 Phosphonate ABC transporter ATP-binding protein		
*nsp18380, phnE4*	PhnE4 Phosphonate ABC transporter permease protein		
**Phosphite transport**			
*nsp35050, ptxA*	*Ptx*A Phosphite ABC transporter permease protein		
*nsp35060, ptxB*	*Ptx*B Phosphite ABC transporter phosphate-binding periplasmic component		
*nsp35070, ptxC*	*Ptx*C Phosphite Phosphite ABC transporter permease protein		
*nsp35080*	phosphite dehydrogenase		
*nsp35090*	*LysR* transcriptional regulator	consistent with the operon structure of the characterised *Pseudomonas stutzeri* phosphite transporter	[118]
**glycerol-3-phosphate transport**			
*nsp7940, ugpC*	glycerol-3-phosphate ATP-binding protein component	no other components of the *ugp* operon appear present	
**P stress inducible**			
*nsp8220, phoH*	PhoH family protein		
**P storage and degradation of P polymers**			
*nsp10230, ppk*	polyphosphate kinase		
*nsp29750, ppa*	inorganic pyrophosphatase		
*nsp42550, ppx*	exopolyphosphatase		
**Degradation of organic P sources**			
*nsp6490*	glycerophophoryl diester phosphodiesterase	contains also a potential phytase domain	
*nsp7010*	atypical alkaline phosphatase	akin to those present in several cyanobacteria	
*nsp12860*	DedA-like phosphatase		
*nsp12920*	alkaline phosphatase		
*nsp12940*	PhoX-like phosphatase		
*nsp18960*	putative PhoX phosphatase		
*nsp20770*	COG4246 superfamily	sometimes annotated as a phytase	
*nsp29340*	Metallophophoesterase		
*nsp29350*	Metallophosphoesterase		
*nsp33000*	predicted phosphatase		
*nsp31680*	metal dependent PHP family phosphoesterase		
*nsp35720*	acid phosphatase		
*nsp46480*	metallophosphoesterase (GlpQ-like)		
*nsp53310*	PhoD-like phosphatase		
**Arsenate-related gene orthologs/operons**			
*nsp40*	ArsA		
*nsp1880*	ArsA		
*nsp15480*	ArsA		
*nsp33490, arsR*	regulator of arsenate resistance		
*nsp33500*	SphX periplasmic P binding component of P ABC transporter		
*nsp33510*	glyceraldehyde-3-phosphate dehydrogenase		
*nsp33520*	major facilitator superfamily permease		
*nsp33540, acr3*	Acr3 (ArsB) Arsenical-resistance protein ACR3		
*nsp33550, arsH*	ArsH Arsenic-resistance protein		
*nsp41360*	ArsC-family protein	ArsC similarity not obvious	
**Haloacid dehalogenase-like hydrolases**			
*nsp1610*	HAD-superfamily hydrolase		
*nsp3740*	HAD-superfamily hydrolase		
*nsp6980*	HAD-superfamily hydrolase		
*nsp48160*	glycoside hydrolase/HAD-superfamily hydrolase		
**P sensing and regulation**			
*nsp10800, phoB*	PhoB (SphR) Response regulator		
*nsp10810, phoR*	PhoR (SphS) sensor kinase		
*nsp10830, phoU*	PhoU putative negative regulator of the Pi regulon		

*N. spumigena* possesses extensive P acquisition machinery and strong TSS were mapped for most of the genes involved. *N. spumigena* CCY9414 contains two copies of a gene encoding a low affinity permease for inorganic phosphate (Pi) transport akin to the *E*. *coli* PitA system (*nsp1550* and *nsp16870*) unlike most marine picocyanobacteria which lack this capacity for P acquisition [102]. In addition, as is the case with several freshwater cyanobacteria [100], [106], the genome of *N. spumigena* CCY9414 contains two gene clusters encoding components of the high affinity Pi transport system. This transport system is comprised of components of the membrane bound ABC transport system (PstABC) and the periplasmic binding protein (PstS) (Table 6). These two high affinity systems appear genetically similar to those characterized biochemically in the freshwater cyanobacterium *Synechocystis* sp. PCC 6803 and may equate to P_i_ ABC transporters with significant differences in both kinetic and regulatory properties [106]. Together, these low and high affinity P_i_ acquisition systems might allow *N. spumigena* to acquire inorganic phosphate over a wide range of concentrations. Other potential high affinity periplasmic P_i_ binding proteins are also encoded in the *N. spumigena* CCY9414 genome similar to *sll0540* (*nsp15300*) and *sll0679* (*nsp33500*) from *Synechocystis* sp. PCC 6803. The latter encodes a variant of the PstS binding protein termed SphX [107], [108], which appears to be regulated differently from the other ‘classic’ PstS proteins at least in *Synechocystis* [106].

In *N. spumigena* CCY9414, *nsp33500* is located in a cluster of genes, *nsp33490–nsp33550* (Table 6) that includes one gene encoding glyceraldehyde-3-phosphate dehydrogenase, but also several others that are all involved in resistance to arsenic acid. Arsenate (As[V]), a toxic P_i_ analog, has a nutrient-like depth profile in seawater [109] and competes with P_i_ for uptake through the PstSCAB system. The gene *nsp33490* encodes a potential ArsR regulator of arsenate resistance and *nsp33540* (ACR3/ArsB) encodes a putative arsenite efflux system (*nsp33550* encodes a putative ArsH but the function of this protein is unknown). *N. spumigena* also encodes three separate copies of genes (*nsp40, nsp1880* and *nsp15480*) potentially encoding ArsA, an arsenite-stimulated ATPase thought to allow more efficient arsenite efflux through ArsB [110], [111]. However, ArsC encoding arsenate reductase appears to be lacking in the *N. spumigena* CCY9414 genome, although an ArsC-family protein is present (*nsp41360*) which may fulfill the role of arsenate reduction.

In addition to transport systems for P_i_ (i.e. phosphorus in its most oxidized form, +5 valence), *N. spumigena* CCY9414 also contains transport systems for phosphonates and phosphite (i.e. +3 valence phosphorus compounds) (Table 6). Transport capacity for these phosphorus sources has only been found in the genomes of some cyanobacteria [99], [100], [112], hence, the presence of transporters for phosphonates and phosphite in *N. spumigena* is intriguing. Phosphonates, organic phosphorus compounds containing a C-P linkage, require a specific C-P lyase enzyme to break this stable bond. In *Pseudomonas stutzeri* and *E*. *coli*, phosphonate utilization is mediated by a cluster of 14 genes (*phnC* to *phnP*) encoding a C–P lyase pathway [113], [114]. The *N. spumigena* CCY9414 genome contains *phnC*-*phnM* (*nsp7450–nsp7590*), with *phnCDE* encoding potential components of a high affinity ABC transport system for phosphonates (there is another copy of *phnE* in this cluster which we have named *phnE3*) and *phnG*-*phnM* encoding the putative membrane-bound C-P lyase complex. In *E*. *coli phnF* and *phnN*-*O* are not required for phosphonate utilization but may encode accessory proteins of the C-P lyase or be transcriptional regulators [113], hence their absence in the *N. spumigena* CCY9414 genome does not preclude the cluster encoding a functional C-P lyase and phosphonate transporter. The *N. spumigena* CCY9414 genome also contains two other gene clusters (*nsp18360–nsp18380* and *nsp35120–nsp35160*) potentially encoding phosphonate ABC transporter components (Table 6), although the latter cluster also contains a truncated *phnH* linked to the *phnM* component of the C*–*P lyase. The role of these clusters in phosphonate utilisation by *N*. *spumigena* remains to be determined, although it is known that other cyanobacteria can utilize this source of phosphorus [115], [116]. In addition to C*–*P lyase cleavage enzymes bacteria may also possess other phosphonatases that cleave the C*–*P bond e.g. phosphonoacetaldehyde phosphonohydrolase [117] belonging to the haloacid dehalogenase (HAD) superfamily. Putative members of this family are also found in the *N. spumigena* CCY9414 genome (Table 6).

The putative *N. spumigena* CCY9414 phosphite transport system (genes *nsp35050–nsp35090*, (Table 6) is similar to the well-characterized *ptxABCDE* system from *Pseudomonas stutzeri* [118], with amino acid identities to the corresponding *P*. *stutzeri* proteins ranging from 40*–*62%. In *P*. *stutzeri*, *ptxABC* encode components of a high affinity phosphite transport system, *ptxD* encodes a NAD-dependent phosphite dehydrogenase oxidizing phosphite to phosphate and *ptxE* is a *lysR* family transcriptional regulator. The *ptxABCD* gene cluster was found in *Prochlorococcus* sp. MIT9301 (only 2 of 18 *Prochlorococcus* genomes currently available possess this cluster) and this was concomitant with the ability of this strain to utilise phosphite as sole phosphorus source [119]. Although the concentration of phosphite in marine waters is unknown the potential obviously exists for *N*. *spumigena* to supplement its phosphorus demand by utilizing this +3 valence phosphorus form.

Further bioinformatic evidence suggestive of the critical nature of phosphorus in the biology of *N*. *spumigena* is the plethora of genes coding for phosphatases that can be found in the genome encompassing over a dozen different gene products, presumably for degradation of organic phosphorus sources (**Table 6**). These genes include an atypical alkaline phosphatase (*nsp7010*) found in several other cyanobacteria [102], [120], putative PhoX phosphatases (*nsp12940* and *nsp19860*) (see [121]), an acid phosphatase (*nsp35720*), and several metallophosphoesterases (*nsp29340, nsp29350, nsp46480*). The product of gene *nsp6490* contains two GlpQ domains and a phytase domain. The former corresponds to the glycerophosphodiester phosphodiesterase domain (GDPD) present in a group of putative bacterial and eukaryotic glycerophosphodiester phosphodiesterases (GP-GDE, EC 3.1.4.46) similar to *E*. *coli* periplasmic phosphodiesterase GlpQ [122], as well as plant glycerophosphodiester phosphodiesterases (GP-PDEs), all of which catalyze the Ca^2+^-dependent degradation of periplasmic glycerophosphodiesters to produce sn-glycerol-3-phosphate (G3P) and the corresponding alcohols. Phytase is a secreted enzyme which hydrolyses phytic acid (the dominant source of phosphorus in soils) to release inorganic phosphate, reinforcing the idea that *N*. *spumigena* is very well equipped to access an array of potential organic, as well as inorganic, P sources in its environment.

### Secondary Metabolites: a Multitude of Biosynthetic Pathways

**Figure 4:**
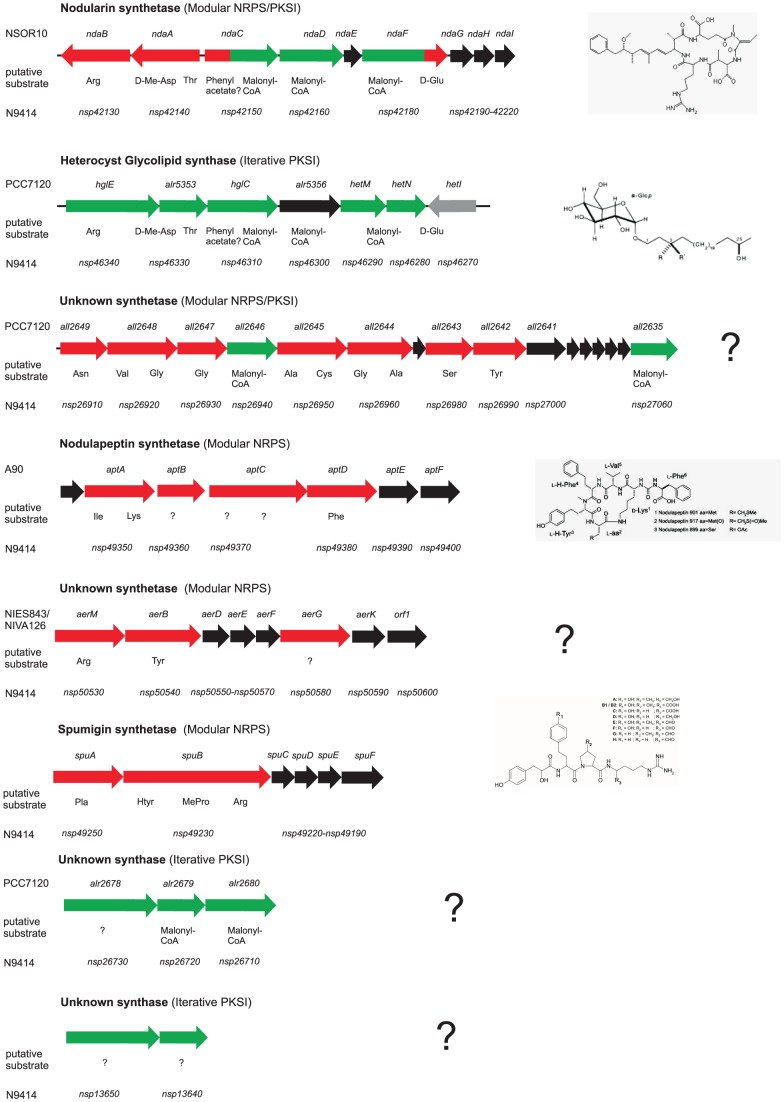
Gene clusters for secondary metabolite biosyntheses in *N. spumigena* CCY9414. The assignment of the gene products to non-ribosomal peptide synthetases (NRPS) or polyketide synthases (PKS) is indicated by red and green colour, respectively. Genes encoding putative tailoring proteins are indicated in black. The classification of PKS into iterative and modular PKS is shown in the subtitle of each gene cluster. For characterized gene clusters product names were included in the subtitles. Related gene clusters if present in the database are shown with their gene annotations and strain names above each gene cluster. Substrate specificities as predicted by http://www.nii.res.in/nrps-pks.html are shown underneath each NRPS or PKS gene containing substrate activating domains. Question marks indicate domains with unclear substrate specificities. The numbers in the second line below the gene clusters relate to the gene numbers in *N. spumigena* CCY9414.

*N. spumigena* CCY9414 produces nodularin, a potent hepatotoxin comprising a cyclic pentapetide containing unusual non-proteinogenic amino acids [10] that is responsible for the deaths of domestic and wild animals throughout the world [10], [123]. Nodularin is synthesized by a hybrid nonribosomal peptide synthetase (NRPS)/polyketide synthase (PKS) enzyme complex [124], as are the heptapeptide hepatotoxic microcystins of freshwater cyanobacteria [125]. The complete nodularin synthetase (*nda*) gene cluster was elucidated from an Australian *N. spumigena* strain [124]. *N. spumigena* CCY9414 contains the nodularin synthetase gene cluster (*nsp42130–nsp42220*), where the order of the genes in the operon and its length, 48 kb, is identical to the Australian isolate (**Fig. 4**).

Investigation of *N. spumigena* strain AV1 from the Baltic Sea led to the discovery of cyclic nodulapeptin peptides and linear spumigin peptides in addition to nodularin [126]. The majority of isolated strains and of trichomes analyzed from the pelagic Baltic Sea are identified as *N. spumigena* [34], [127], [128] and contain nodularin as well as spumigins and nodulapeptins [129]. Peptide synthetase gene clusters encoding the biosynthetic pathways for the production of spumigins (*nsp49190–nsp49250*) and nodulapeptins (*nsp49350–nsp49400*) were identified in the genome of *N. spumigena* CCY9414 [130], [131] (**Fig. 4**).

Surprisingly, analysis of the genome identified gene clusters for one additional NRPS, two additional PKS and one additional hybrid NRPS/PKS gene cluster encoding unknown peptides (**Fig. 4**). A compact NRPS gene cluster (*nsp50530–nsp50600*) consisting of 3 modules and proteins encoding the biosynthesis of a 2-carboxy-6-hydroxyoctahydroindole moiety (Choi) was identified suggesting that *N. spumigena* CCY9414 might produce an aeruginosin (**Fig. 4**). Aeruginosins are linear tetrapeptide protease inhibitors found in the genera *Planktothrix* and *Microcystis* [132], [133] but which have never been reported from *N. spumigena*. Additionally, a large cryptic NRPS-PKS gene cluster (*nsp26910–nsp27060*) was found (**Fig. 4**). The product is not known, but a very similar gene cluster is present in *Anabaena* PCC 7120. It is interesting to note that the gene clusters for nodularin, spumigin, nodulapeptin and the cryptic gene cluster that is supposed to make aeruginosin are not randomly distributed but cluster in a 0.8 Mb region of the genome.

In addition to the PKS modules that were identified as part of non-ribosomal peptide synthetase (NRPS) gene clusters, two further PKS gene clusters (*nsp26710–nsp26730* and *nsp13640–nsp13650*) were discovered in the genome of *N. spumigena* CCY9414 (**Fig. 4**). Unlike the modular PKS, these enzymes comprise up to three consecutive acyl carrier protein domains (ACPs, data not shown), indicating their involvement in an iterative fatty acid like mode of biosynthesis [134]. The classification as iterative PKS is further supported by their phylogenetic clustering in an overall phylogenetic tree of PKS sequences ([135], data not shown). Two closely related heterocyst glycolipid synthases (*nsp13650* and *nsp46340*) were also identified (**Fig. 4**). One of the clusters shows close similarity to the heterocyst glycolipid biosynthesis clusters of *Anabaena* PCC 7120 [136] and is most likely involved in the biosynthesis of this important heterocyst envelope compound. Structure-based models allow the prediction of the substrate for the acyltransferase (AT) domain of PKS proteins (http://www.nii.res.in/nrps-pks.html). Using this specificity conferring software it was predicted that the two uncharacterized PKS could be involved in the synthesis of unusual (e.g. branched) fatty acids. One of the clusters is also present in *Anabaena* PCC 7120. The structure and role of these unusual lipids is unknown.

Cyanobacteria are increasingly recognized as a source of a second class of peptidic natural products that are produced through the post-translational modification of precursor proteins. Three different peptide families, cyanobactins [137], [138], microviridins [139], [140] and lantipeptides (prochlorosins) [141] have been described and differ substantially in their respective amino acid functionalities and mode of macrocyclization. The genetic information for the production of two of these classes, cyanobactin (*nsp33610–nsp33660*) and microviridin (*nsp49400–nsp49480*) is present in the *N. spumigena* CCY9414 genome [139]. However, the PatA homolog encoded in the cyanobactin cluster of *N. spumigena* CCY9414 is truncated and the cluster lacks a precursor gene, most likely rendering the gene cluster non-functional. The *N. spumigena* CCY9414 genome further features 7 cryptic bacteriocin gene clusters although none encodes the LanM enzyme, which characterizes the lantipeptide family [142], and two gene clusters related to sunscreen biosynthesis.

Genomic mining approaches and subsequent *in vitro* reconstitution studies have previously uncovered the biosynthetic pathways for two important sunscreen compounds in cyanobacteria, mycosporic acids (MAA) and scytonemin [143*]*–[145]. Both compounds show a sporadic distribution in cyanobacteria and are predominantly detected in terrestrial and microbial mat communities [146]. The fact that both biosynthesis gene clusters are present in the genome of the brackish water *N. spumigena* CCY9414 was therefore unexpected and may give some new implications for the specific adaptation to the brackish water environment as well as the capability to form surface scums.

Summarizing, NRPS and PKS comprise at least 4% of the genome of *N. spumigena* CCY9414. This number includes 9 gene clusters encoding 58 genes and occupying 222 kb of the genome. This is more than the 3% reported for *Moorea producens* (*Lyngbya majuscula* 3L), one of the most prolific sources of natural metabolites among cyanobacteria [147]. Thus, the genetic information required for the generation of these secondary metabolites takes a substantial part of the genomic coding capacity. Even though *N. spumigena* is the subject of frequent chemical analysis, the only other secondary metabolites observed were nodularin, nodulapeptins and spumigins [126], [130], [148]. This genome analysis suggests that *N. spumigena* has the potential to synthesise a wealth of other peptides and polyketides. There is still enormous interest in new bioactive compounds from bacteria and their biosynthetic pathways. Many bacterial NRPS and PKS products have served as lead products for drug development and the information gained on NRPS and PKS can provide new insights for the generation of “unnatural” compound libraries by combinatorial biosynthesis approaches (e.g. [149]). Another, new class of bioactive compounds in cyanobacteria are ribosomally produced and posttranslationally modified peptides [150]. In order to use the potential of this *N. spumigena* strain in the future, genomic mining strategies have to be developed in order to identify the secondary metabolites guided by the substrate predictions for the synthesizing enzymes.

## Strains and Methods

### Ethic Statement

This research did not involve endangered or protected species and no work on vertebrates. The microbial sampling was done on board of a German research vessel (FS Alkor, Institute of marine Sciences, Kiel) that had all the permissions to sample in the Baltic Sea waters. The Bornholm Sea is neither a marine park nor private property.

*N. spumigena* CCY9414 was isolated from samples collected from the surface water in the Bornholm Sea by picking single aggregates of trichomes and plating on agar medium of a mixture of 1 part ASN3 and 2 parts BG11, devoid of combined nitrogen [33]. The isolated strain *N. spumigena* CCY9414 is a toxic planktonic, heterocyst-forming, gas-vacuolate bloom-forming cyanobacterium and is representative of those *N. spumigena* that form toxic surface blooms in brackish coastal seas.

### Genome analysis

The genome was sequenced using a combination of Sanger and 454 sequencing platforms. For Sanger sequencing, two genomic libraries with insert sizes of 4 and 40 kb were made. The prepared plasmid and fosmid clones were end-sequenced to provide paired-end reads at the J. Craig Venter Science Foundation Joint Technology Center on ABI 3730XL DNA sequencers (Applied Biosystems, Foster City, CA). Whole-genome random shotgun sequencing produced 47,486 high quality reads averaging 811 bp in length, for a total of approximately 38.5 Mbp of DNA sequence, analysed as described [151] and leading to the 5.32 Mb Whole Genome Shotgun Assembly deposited in GenBank under the accession number PRJNA13447. For this assembly, 4,904 genes, among them 4,860 protein-coding genes were predicted.

Since it was not possible to get a single large scaffold from Sanger sequencing reads alone, and because several previously analysed genes were missing, additional sequence data was obtained by pyrosequencing using the GS FLX system provided by Eurofins MWG GmbH Ebersberg, Germany. The GS FLX system delivered 109,881 sequence reads with an average read length of 251 base pairs. A hybrid 454/Sanger assembly was made using the MIRA assembler [152]. Resulting contigs were joined into scaffolds using BAMBUS [153]. Altogether, an average 13-fold coverage of the genome was obtained. Gene calling and initial annotation was performed applying the Rapid Annotations using Subsystems Technology (RAST) system [154], leading to the Whole Genome Shotgun Assembly deposited at DDBJ/EMBL/GenBank under the accession AOFE00000000. The version described in this paper is the first version, AOFE01000000.

### Cultivation and RNA Preparation for Transcriptome Analysis

*N. spumigena* CCY9414 cells were grown in cell culture bottles using a 2∶1 mixture of nitrate-free BG11 and - ASN-III media [2] (salinity 10 PSU). Cells were incubated at ambient air in a temperature controlled incubator at 20°C, 40 µmol photons m^−2^ s^−1^. The photoperiod was set at 16 h light and 8 h dark. Cells were mixed by daily shaking of the cell culture bottles. 50 ml of cells from the middle of the light period were harvested by quick filtration through sterile glass fibre filters (Whatman GF/F). Filters and cells were immediately frozen in liquid nitrogen and stored at −80°C.

Total RNA of *N. spumigena* CCY9414 was isolated using the Total RNA Isolation Kit for plants (Macherey-Nagel). To improve RNA yield, ice-cold lysis buffer (buffer RAP, Macherey-Nagel) was added to the frozen cells on filters and the mixture was shaken with steel beads (cell mill MM400, Retsch) with maximum speed, three times for 30 seconds. For sequence analysis, cDNA libraries were constructed (vertis Biotechnologie AG, Germany) and analysed on an Illumina sequencer as previously described [19]. In brief, total RNA was enriched for primary transcripts by treatment with Terminator^™^ 5'phosphate-dependent exonuclease (Epicentre). Then, 5'PPP RNA was cleaved enzymatically using tobacco acid pyrophosphatase (TAP), the 'de-capped' RNA was ligated to an RNA linker [19] and 1^st^-strand cDNA synthesis initiated by random priming. The 2^nd^ strand cDNA synthesis was primed with a biotinylated antisense 5'-Solexa primer, after which cDNA fragments were bound to streptavidin beads.

Bead-bound cDNA was blunted and 3' ligated to a Solexa adapter. The cDNA fragments were amplified by 22 cycles of PCR. For Illumina HiSeq analysis (100 bp read length), the cDNA in the size range of 200 – 500 bp was eluted from a preparative agarose gel. A total of 41,519,905 reads was obtained. The data was deposited in the NCBI Short Read Archive under accession SRS392745.

Reads were mapped to the genome using segemehl [155] with default settings, resulting in 40,577,305 mapped reads. Transcriptional start sites (TSSs) were predicted for positions where ≥280 reads start and the number of reads starting at the position is ≥50% larger than the number of reads covering the position. Classification of TSSs into gTSSs, iTSSs, aTSSs and nTSSs was carried out according as described [19].

### Data Interpretation

Protein sequences were compared with those from *Anabaena variabilis* ATCC 29413, *Anabaena* PCC 7120, *Nostoc punctiforme* PCC 73102 and *Synechocystis* sp. PCC 6803 using BLASTp with an e-value cut-off of 1e^−8^. High scoring sequence pairs for the same sequences were merged and the per cent identity and alignment length values recomputed. Merged high scoring sequence pairs with alignment length coverage less than 10% of the longer sequence were removed. Those sharing the same query or subject sequence were filtered as follows: first, the best hit was kept together with hits whose per cent identity is at most ten percentage points smaller; second, we removed those hits whose alignment length coverage was more than 20 percentage points smaller than that of the best hit. The remaining hits were clustered using MCL with default parameters. Based on this clustering we defined unique and shared genes of the genomes. Phylogenetic classification of protein sequences was carried out using MEGAN. BLASTp results against the NCBI nr database requiring a minimum e-value of 1e^−8^ were used as input.

IS elements were identified and assigned to IS families based on the genes or gene fragments encoding transposase by the ISfinder algorithm [26] using default parameters and a BLASTp threshold of E≤1e^−5^.

### Analysis of secondary metabolite genes

NRPS and PKS gene clusters gene clusters were identified using met2db [156]. Adenylation domain substrate specificity predictions for NRPS enzymes were made using NRPSpreditor2 [157]. Catalytic domain annotations for NRPS and PKS proteins were refined manually using CD-search, BLASTP and InterProScan. Putative functions were assigned to proteins encoding tailoring enzymes associated with these cluster were also identified using CD-search, BLASTP and InterProScan searches. The cyanobactin gene cluster was identified using sequences from the patellamide gene cluster as a query in BLASTp searches.

## Supporting Information

Figure S1 **Cluster analysis of proteins potentially involved in sucrose metabolism in cyanobacteria.** Putative proteins from *N. spumigena* CCY9414 (labelled *nsp* and in boldface letters) are included. Sps – sucrosephosphate synthase, Spp – sucrosephosphate phosphatase, Sus – sucrose synthase. The evolutionary history was inferred using the Minimum Evolution method within MEGA5 [158]. The optimal tree with the sum of branch length  =  7.8464659 is shown. The percentage of replicate trees in which the associated taxa clustered in the bootstrap test (10,000 replicates) are shown next to the branches if >60. The tree is drawn to scale, with branch lengths in the same units as those of the evolutionary distances used to infer the phylogenetic tree and are in the units of the number of amino acid substitutions per site. All positions with less than 50% site coverage were eliminated. There were a total of 716 positions in the final dataset.
(PPTX)

Figure S2 **Fusion proteins between an IsiA/CP43 homolog and PsaL in** ***Anabaena*** **7120 and** ***N. spumigena*** **CCY9414. **A. Sequence alignment of the CP43-PsaL fusion proteins from *N. spumigena* CCY9414 (*Nsp*37500) and *Anabaena* PCC7120 (All4002) and the respective PsaL proteins (*Nsp*40050 and All0107). B. Prediction of transmembrane helices for the *Nsp*37500 fusion protein (numbered I to IX). The topology and possible transmembrane helices were predicted using TMHMM 2.0 at http://www.cbs.dtu.dk/services/TMHMM/. PsbC-PsaL hybrid proteins similar to *Nsp*37500 exist in only nine other cyanobacteria: in *Anabaena* PCC 7120, *Moorea producens* (*Lyngbya majuscula* 3L), *Leptolyngbya* sp. PCC 7375, *Fischerella* sp. JSC-11, *Trichodesmium erythraeum* IMS101, *Synechococcus* spp. JA-2-3B'a(2–3) and JA-3-3Ab, *Oscillatoria* sp. PCC 6506 and *Crocosphaera watsonii* WH0003.
(PPTX)

Table S1 **Families of IS elements in *N. spumigena* CCY9414.**
(XLSX)

Table S2 **Details of 608 gene clusters that are common to three well-studied Nostocales (Fig. 2A) but not found in *N. spumigena* CCY9414.** The acronyms are as follows: N_punct, *Nostoc punctiforme* sp. PCC 73102; A_var, *Anabaena variabilis* sp. ATCC 29413; N_7120, *Anabaena* PCC 7120, based on MCL clustering of BLASTp results (minimum e-value: 10^−8^).
(XLSX)

Table S3 **List of predicted *N. spumigena* CCY9414 proteins not present in *Anabaena* PCC 7120, *Nostoc punctiforme* sp. PCC 73102, or *Anabaena variabilis* sp. ATCC 29413.**
(XLSX)
