# Supplementary material for: Insights into the Physiology and Ecology of the Brackish-Water-Adapted Cyanobacterium Nodularia spumigena CCY9414 Based on a Genome-Transcriptome Analysis
Source: PLoS One. 2013 Mar 28;8(3):e60224. doi: 10.1371/journal.pone.0060224 (PMC3610870; doi:10.1371/journal.pone.0060224)
Supplement: Figure S1 — Cluster analysis of proteins potentially involved in sucrose metabolism in cyanobacteria. Putative proteins from N. spumigena CCY9414 (labelled nsp and in boldface letters) are included. Sps – sucrosephosphate synthase, Spp – sucrosephosphate phosphatase, Sus – sucrose synthase. The evolutionary history was inferred using the Minimum Evolution method within MEGA5 [158]. The optimal tree with the sum of branch length = 7.8464659 is shown. The percentage of replicate trees in which the associated taxa clustered in the bootstrap test (10,000 replicates) are shown next to the branches if >60. The tree is drawn to scale, with branch lengths in the same units as those of the evolutionary distances used to infer the phylogenetic tree and are in the units of the number of amino acid substitutions per site. All positions with less than 50% site coverage were eliminated. There were a total of 716 positions in the final dataset. (PPTX) [file pone.0060224.s001.pptx]

## Slide 1
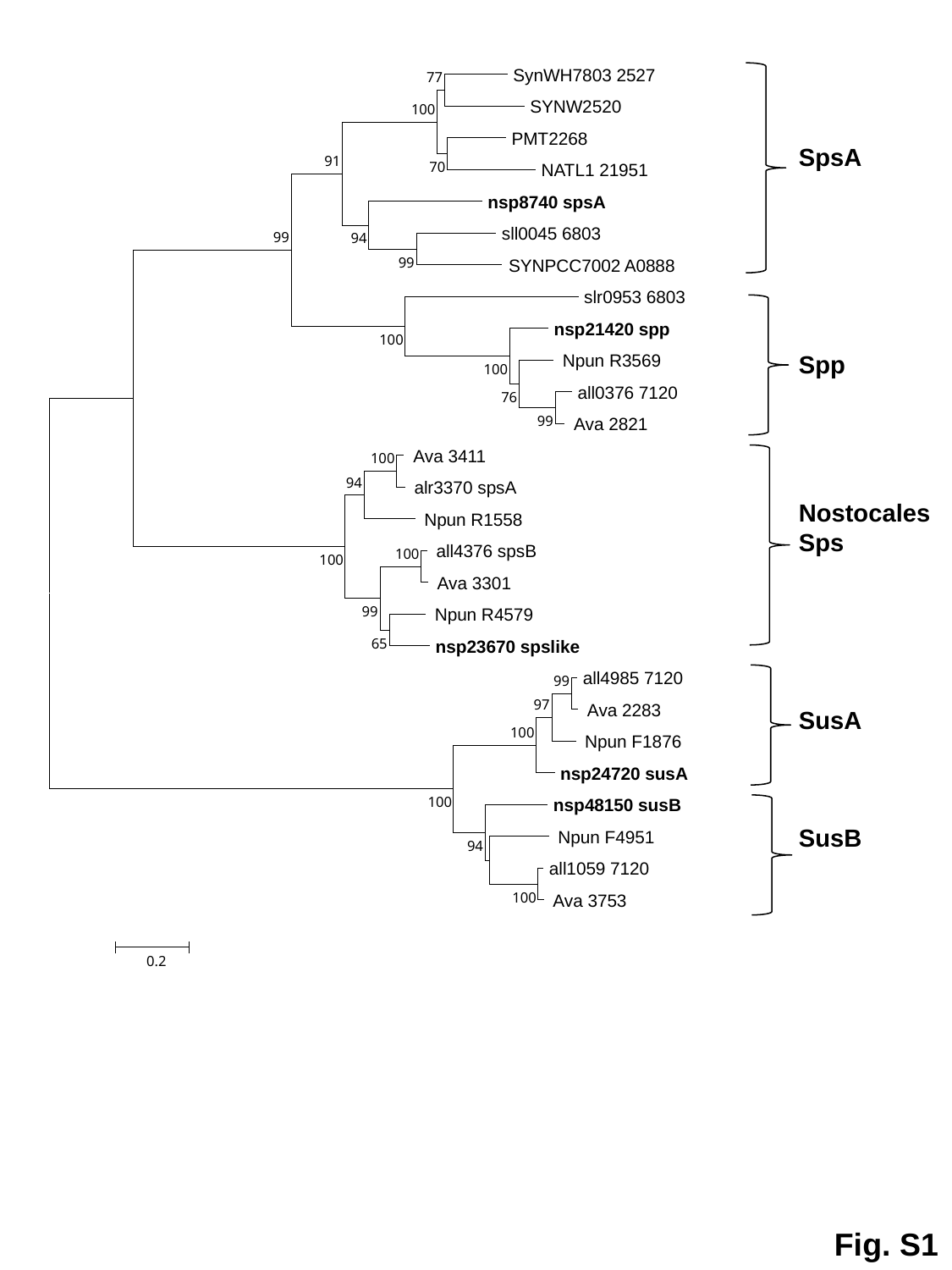

SynWH7803 2527
77
 SYNW2520
100
 PMT2268
91
70
 NATL1 21951
 nsp8740 spsA
 sll0045 6803
99
94
99
 SYNPCC7002 A0888
 slr0953 6803
 nsp21420 spp
100
 Npun R3569
100
 all0376 7120
76
99
 Ava 2821
 Ava 3411
100
94
 alr3370 spsA
 Npun R1558
 all4376 spsB
100
100
 Ava 3301
99
 Npun R4579
65
 nsp23670 spslike
 all4985 7120
99
97
 Ava 2283
100
 Npun F1876
 nsp24720 susA
100
 nsp48150 susB
 Npun F4951
94
 all1059 7120
100
 Ava 3753
0.2
SpsA
Spp
Nostocales
Sps
SusA
SusB
Fig. S1
