# Supplementary figures and images for: Insights into the Physiology and Ecology of the Brackish-Water-Adapted Cyanobacterium Nodularia spumigena CCY9414 Based on a Genome-Transcriptome Analysis
Source: PLoS One. 2013 Mar 28;8(3):e60224. doi: 10.1371/journal.pone.0060224 (PMC3610870; doi:10.1371/journal.pone.0060224)

## Slide 1
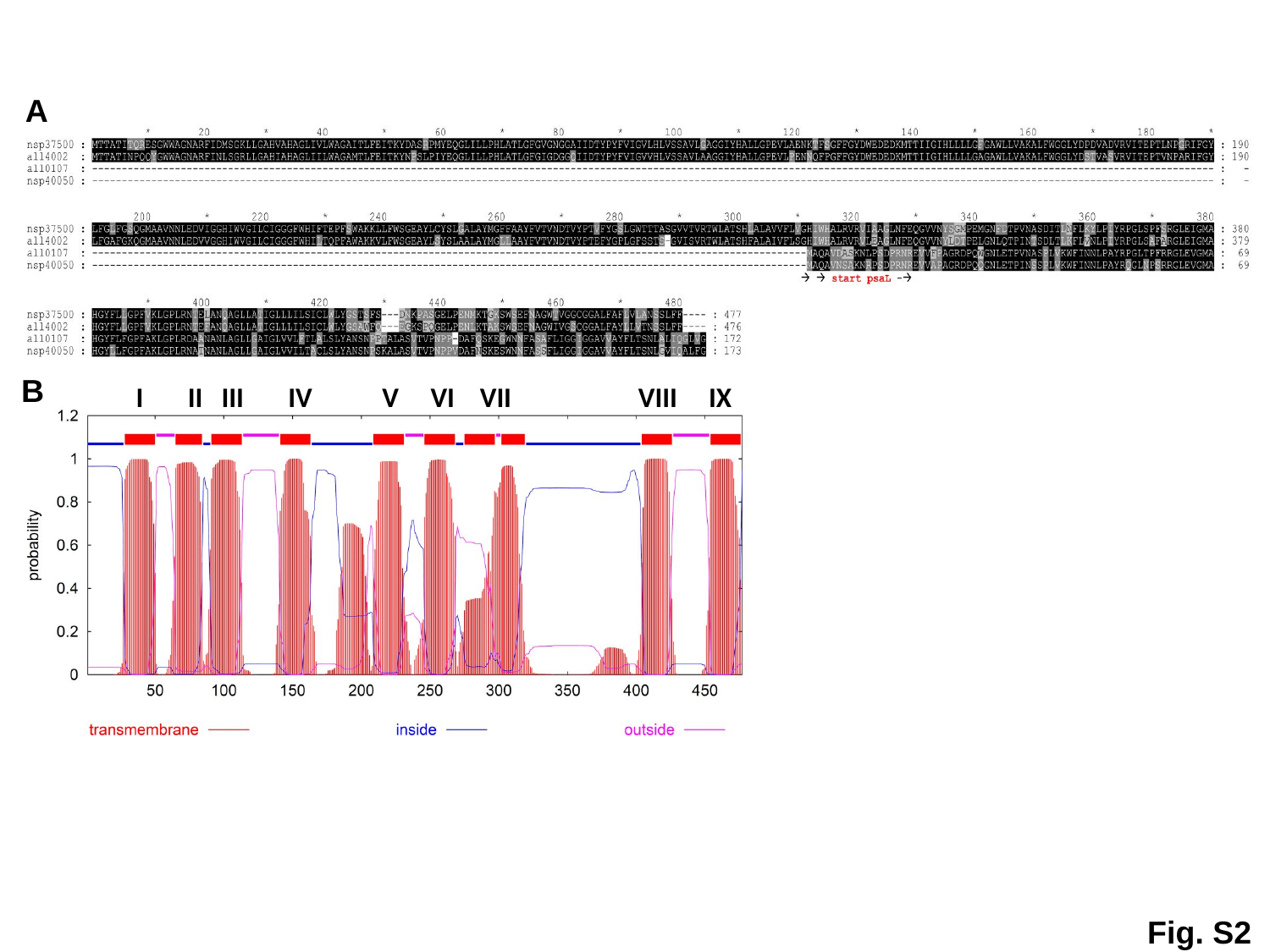

A
B
I II III IV V VI VII VIII IX
Fig. S2

Supplement: Figure S2 — Fusion proteins between an IsiA/CP43 homolog and PsaL in Anabaena 7120 and N. spumigena CCY9414. A. Sequence alignment of the CP43-PsaL fusion proteins from N. spumigena CCY9414 (Nsp37500) and Anabaena PCC7120 (All4002) and the respective PsaL proteins (Nsp40050 and All0107). B. Prediction of transmembrane helices for the Nsp37500 fusion protein (numbered I to IX). The topology and possible transmembrane helices were predicted using TMHMM 2.0 at http://www.cbs.dtu.dk/services/TMHMM/. PsbC-PsaL hybrid proteins similar to Nsp37500 exist in only nine other cyanobacteria: in Anabaena PCC 7120, Moorea producens (Lyngbya majuscula 3L), Leptolyngbya sp. PCC 7375, Fischerella sp. JSC-11, Trichodesmium erythraeum IMS101, Synechococcus spp. JA-2-3B'a(2–3) and JA-3-3Ab, Oscillatoria sp. PCC 6506 and Crocosphaera watsonii WH0003. (PPTX) [file pone.0060224.s002.pptx]
